# Impact of different ionization states of phosphorylated Serine-65 on ubiquitin structure and interactions

**DOI:** 10.1038/s41598-018-20860-w

**Published:** 2018-02-08

**Authors:** Yaniv Kazansky, Ming-Yih Lai, Rajesh K. Singh, David Fushman

**Affiliations:** Department of Chemistry and Biochemistry, Center for Biomolecular Structure and Organization, University of Maryland, College Park, MD 20742 USA

## Abstract

The covalent attachment of ubiquitin (Ub) or Ub chains to cellular proteins is a versatile post-translational modification involved in a variety of eukaryotic cellular events. Recently, the post-translational modification of Ub itself by phosphorylation has emerged as an important component of the Ub-signaling system. Specifically, Ub phosphorylation at serine-65 was shown to activate parkin-mediated mitochondrial quality control. However, the impact of phosphorylation on Ub structure and interactions is poorly understood. Here we investigate the recently reported structural changes in Ub upon serine-65 phosphorylation, namely, the equilibrium between a native-like and a novel, alternate conformer of phosphorylated Ub (pUb). We show that this equilibrium is pH-dependent, and the two pUb conformers are linked to the different charge states of the phosphate group. We examined pUb binding to a known Ub-receptor and found that the alternate conformer is binding incompetent. Furthermore, serine-65 phosphorylation affects the conformational equilibrium of K48-linked Ub dimers. Lastly, our crystal structure of S65D Ub and NMR data indicate that phosphomimetic mutations do not adequately reproduce the salient features of pUb. Our results suggest that the pH-dependence of the conformations and binding properties of phosphorylated Ub and polyUb could provide an additional level of modulation in Ub-mediated signaling.

## Introduction

The covalent attachment of ubiquitin (Ub) or Ub chains (polyUb) to cellular proteins is one of the most versatile known post-translational modifications, with numerous signals being encoded by different types of Ub-Ub linkages^1^. Recently, Ub phosphorylation at S65 has come to light as a novel and important component in the PINK1 (PTEN-induced novel kinase protein 1)-Parkin pathway. Parkin is an E3 Ub ligase^2,3^ believed to have a significant role in mitochondrial quality control^3–6^, and defects in the PINK1-Parkin pathway have been linked to autosomal recessive forms of early-onset Parkinson’s Disease^5,7–10^. In its native conformation, parkin is autoinhibited^11–16^, and phosphorylation of both parkin and Ub by PINK1 at a conserved S65 appears to be necessary for complete activation of parkin’s E3 ligase activity^17–22^. In addition to S65, phosphorylation of Ub has been observed at several other residues^23–26^, suggesting that phosphorylated Ub (pUb) may play significant roles within the Ub-signaling system. The roles of Ub phosphorylation at the non-S65 sites and the respective kinases and phosphatases remain unknown. Ub modification by phosphorylation has thus emerged as an important regulatory mechanism of the Ub signaling system that requires rigorous exploration and characterization.

A recent study by Wauer *et al*. of S65 pUb^27^ found evidence of a novel, alternate conformer of Ub, designated here as pUb^alt^, which is in slow exchange (~2 per sec) with the “native-like” conformer, designated as pUb^nat^. The roles and origins of this novel conformer, however, are unclear. Strikingly, there is no evidence of such a conformer in phosphomimetic Ub variants, in which S65 is replaced with aspartate or glutamate, raising interesting questions about the origin of the pUb^alt^ conformer. Since both phosphomimetic mutation and phosphorylation at S65 introduce a negative charge at the same position of Ub, we asked the question of what unique properties resulting from phosphorylation could result in the pUb^alt^ conformer.

Here, we put forth and test two hypotheses to understand the origins of this conformer: (1) that it is an NMR artifact resulting from Ub oligomerization, and (2) that it results from a (−2) charge on the phosphate group. To test these hypotheses we analyzed temperature and pH-dependence of both pUb conformers, determined their spin-relaxation rates, and characterized and compared their ability to bind to a known Ub-receptor with the binding properties of wild-type Ub (Ub^WT^) and phosphomimetic S65D Ub (Ub^S65D^) variant. We also present the first crystal structure of Ub^S65D^, which reveals the structural effects of introducing a single negative charge at position 65 in Ub. Given that phosphorylated Ub chains are found in cells^28,29^, understanding how Ub phosphorylation affects the structure of polyUb chains is critical to a complete understanding of the effect of this modification. Therefore, we also examined the effect of Ub phosphorylation on the conformations of Ub dimers linked via K48, the most abundant linkage in cells. Our results suggest that the singly- and doubly-deprotonated states of phosphorylated S65 have differential effect on Ub structure and interactions, and that phosphomimics are poor structural models for pUb.

## Results

**Figure 1:**
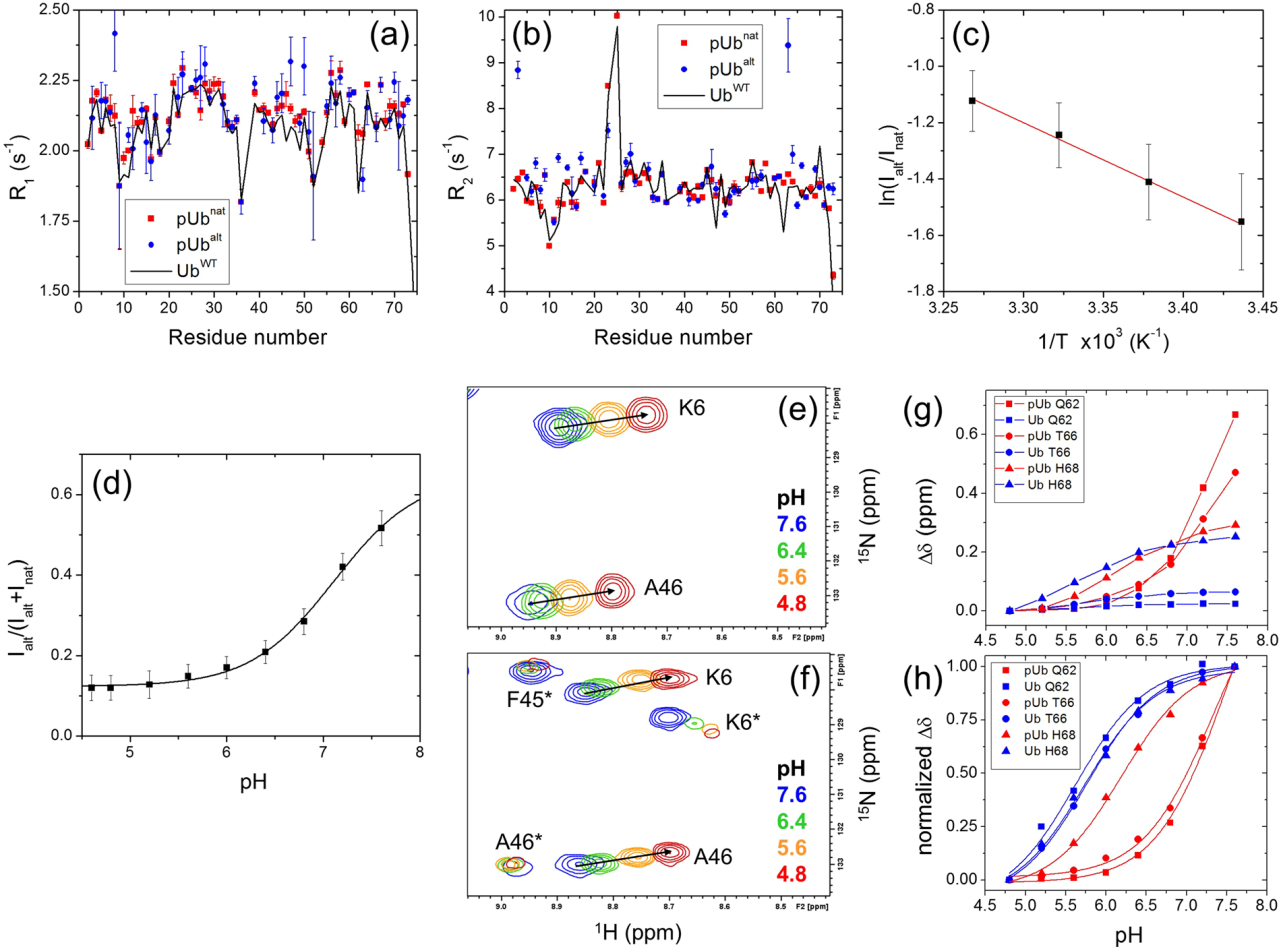
NMR characterization of the two conformers of pUb. (**a**,**b**) Longitudinal (R_1_) and transverse (R_2_) ^15^N relaxation rates for the pUb^nat^ (red) and pUb^alt^ (blue) signals of pUb, as well as for Ub^WT^ (black line). (**c**) Temperature dependence of the ratio of signal intensities, *I*_alt_ and *I*_nat_, of the pUb^alt^ and pUb^nat^ conformers, averaged over 16 residues (the symbols and error bars represent the mean and the standard deviation). The line represents a linear regression fit to the Boltzmann distribution of the relative populations of the two conformers, see Equation (1) (Methods). (**d**) The ratio of signal intensities of the two pUb conformers representing the relative population of pUb^alt^, as a function of pH. The ratio was averaged over 27 residues, the error bars represent standard deviations. The line represents the fit of these data to Equation (2) (see Methods). (**e**,**f**) Representative regions of the ^1^H-^15^N correlation spectra of Ub^WT^ (**e**) and pUb (**f**) showing signal shifts for select residues as a function of pH. The asterisks in F indicate pUb^alt^ signals. (**g**,**h**) CSPs (**g**) and normalized CSPs (**h**) as a function of pH for the indicated residues in Ub^WT^ and pUb^nat^. The curves in (**h**) represent the results of fit of the normalized CSPs to Equation (3) (Methods). The pK_a_ values extracted from the CSPs of Q62, T66, and H68 signals are 5.6, 5.8, and 5.7, respectively, in Ub^WT^ and 7.5, 7.3, and 6.2 for the signals of the same residues in pUb^nat^.

After purifying phosphorylated Ub (see Methods), we recorded a ^1^H-^15^N NMR correlation spectrum of the protein and observed the additional signals reported by Wauer *et al*.^27^ (Figure S1a). Shifts in NMR signals reflect a change in the electronic environment of nuclei which could reflect an altered 3D structure inside Ub but could also be caused by “external” factors such as intermolecular interactions. Note that the residues exhibiting strongly shifted alternate signals in pUb are located at or near the hydrophobic patch on Ub surface comprising residues L8, I44, and V70 (Figure S1f). Because a negative charge near the hydrophobic patch surface can promote interactions between a Ub-like domain and Ub^30^ (and by extension between two Ubs), which could subsequently affect chemical shifts of the residues at or near the interface, we tested whether the additional signals observed upon S65 phosphorylation could be a result of noncovalent Ub oligomerization upon the attachment of the phosphoryl group. To this end, we determined longitudinal (R_1_) and transverse (R_2_) ^15^N relaxation rates for backbone amides in pUb and compared the results for the “native-like” and the “alternate” NMR signals. The formation of a Ub dimer is expected to increase the R_2_ and decrease the R_1_ by approximately 1.6 fold^31^. Our results show essentially no difference between the two groups of signals (Fig. 1a and b) and therefore no evidence of oligomerization. This rules out Ub:Ub interactions as a possible cause of the observed pUb^alt^ conformer. Furthermore, the similarity of the transverse relaxation rates for both Ub conformers allowed us to use intensities of the corresponding NMR signals in order to quantify the relative populations of pUb^nat^ and pUb^alt^ in the subsequent analysis.

The ^15^N relaxation rates in pUb are generally similar to those in Ub^WT^ (Fig. 1a and b), both in the overall level and the residue-specific variations, indicating that the rotational diffusion (hence the shape) and the local backbone motions characteristic for Ub are preserved in pUb. Interestingly, several residues located near the phosphorylation site (most notably, I3 and K63) exhibited elevated R_2_ values in pUb^alt^, suggesting the presence of conformational exchange on the μs-ms time scale in this conformer. In addition, the ^15^N relaxation rates for L73 in pUb^alt^ are markedly higher than in pUb^nat^ and Ub^WT^, consistent with the suggested retraction of the C-terminal tail which would render residues 72–73 less flexible.

### The conformational equilibrium of S65 phosphorylated Ub is controlled by pH

Since the observed pUb^alt^ and pUb^nat^ NMR signals belong to distinct conformational states of pUb, we utilized the temperature dependence of the relative intensities of these signals in order to thermodynamically characterize the equilibrium between the corresponding states. As expected, the difference in the relative populations of pUb^alt^ and pUb^nat^ decreased with temperature. The linear dependence of the natural logarithm of the populations ratio versus 1/T in the temperature range from 291 to 306 K (Fig. 1c) allowed us to extract thermodynamic parameters of this equilibrium: ΔH = 22.0 ± 5.8 kJ/mol, ΔS = 62.5 ± 18.9 J/(mol·K), and ΔG = 3.32 ± 0.31 kJ/mol at 25 °C.

Because the covalently attached phosphate group can be singly or doubly deprotonated (having the (−1) or (−2) charge, respectively), we also hypothesized that the two observed Ub conformers could be coupled to the charge state of the phosphate group on S65. To test if the charge state of the phosphate controls the equilibrium between pUb^nat^ and pUb^alt^, we prepared pUb in a citric acid-phosphate buffer at pH 7.6 and then gradually decreased the pH down to 4.6, recording NMR spectra at every titration point. We observed a systematic decrease in the intensity of the pUb^alt^ signals over the course of the pH titration: these signals were comparable in intensity to the corresponding pUb^nat^ signals at pH 7.6 but almost vanished at pH 4.6. This decrease of the pUb^alt^ signals at low pH was not caused by a loss of the phosphoryl group, as evident from the absence of the signal of unphosphorylated S65 in the pUb spectrum at pH 4.8 (Figure S2c,d) indicating that the protein was still entirely in its phosphorylated state. The ratio of signal intensities, *I*_alt_/(*I*_alt_ + *I*_nat_), reflecting the fraction of Ub molecules in the pUb^alt^ conformation, exhibited a sigmoidal behavior as a function of pH (Fig. 1d) which could be fit to a single pK_a_ value of 7.08 ± 0.13 (the individual values for different residues spread from 6.8 to 7.4). This pK_a_ value is consistent with the pK_a2_ (6.9–7.2) of phosphoric acid^32,33^ (related to the equilibrium between dihydrogen and monohydrogen phosphate ions) and somewhat higher than the reported random-coil pK_a_ value (≈6) of pSer^34^. Note that H68, the other Ub residue titratable in the pH range (4.6–7.6) studied here, has a pK_a_ of 5.5^35^ and therefore is not expected to have a significant effect on the pK_a_ values determined above. This suggests that the observed pH dependence of the relative intensities of pUb^alt^ and pUb^nat^ signals (Fig. 1d) primarily reflects the change in the charge/protonation state of the phosphate group on S65.

Our observation of the pH-dependent conformational switch in pUb generally agrees with the report by Dong *et al*.^36^ that appeared online as this manuscript was in the process of submission. The general agreement of our pKa values with those derived in that publication using ^31^P NMR signals of pS65 corroborates our findings.

Interestingly, in addition to the changes in the relative signal intensities of the pUb^nat^ and pUb^alt^ conformers with pH, we observed pH-dependent shifts for many amides in pUb. Notably, the pUb^alt^ signals showed significantly lesser shifts with pH than the pUb^nat^ signals. To examine the effects of phosphorylation on the chemical shifts of pUb, we compared our results with a similar pH titration of Ub^WT^. Both pUb^nat^ and Ub^WT^ signals generally followed a linear trajectory on the ^1^H-^15^N map indicating that the ^1^H and ^15^N resonances are affected by pH proportionally (Figs 1e and f and S2a,b). We examined amide chemical shift perturbations (CSPs) as a function of pH for each residue in order to determine whether the observed effects were caused by the phosphate on S65 or by some other titratable group, such as H68. We found that the CSPs of the residues in the vicinity of H68 (e.g. K6, I44, A46, G47, Q49, L69) behaved similarly for both Ub^WT^ and pUb^nat^, and their pH dependence was consistent with the reported pK_a_ of H68 (pK_a_≈5.5^35^). However, residues closer to the phosphorylation site, e.g., Q62 and T66, showed far greater CSPs in pUb compared to Ub^WT^ (Figs 1g and S2a,b). In addition, their normalized CSPs in pUb showed a comparatively larger increase between pH 6.5 and 7.5, in the vicinity of pK_a2_ of phosphate (Fig. 1h), and could be fit with a single pK_a_ value of 7.5 (Q62) and 7.3 (T66). Also the side chain amide signals of Q62 in pUb shifted significantly over the course of the pH titration (pK_a_ = 7.5) whereas those of Ub^WT^ did not move (Figure S2a,b,e). Interestingly, these shifts were strikingly different for the H_ε21_ and H_ε22_ protons, both in the magnitude and the direction, suggesting differential contacts of the two protons with the surrounding atoms. The implications of this will be considered in the discussion section.

### Do phosphomimetic mutations reproduce the effect of phosphorylation?

**Figure 2:**
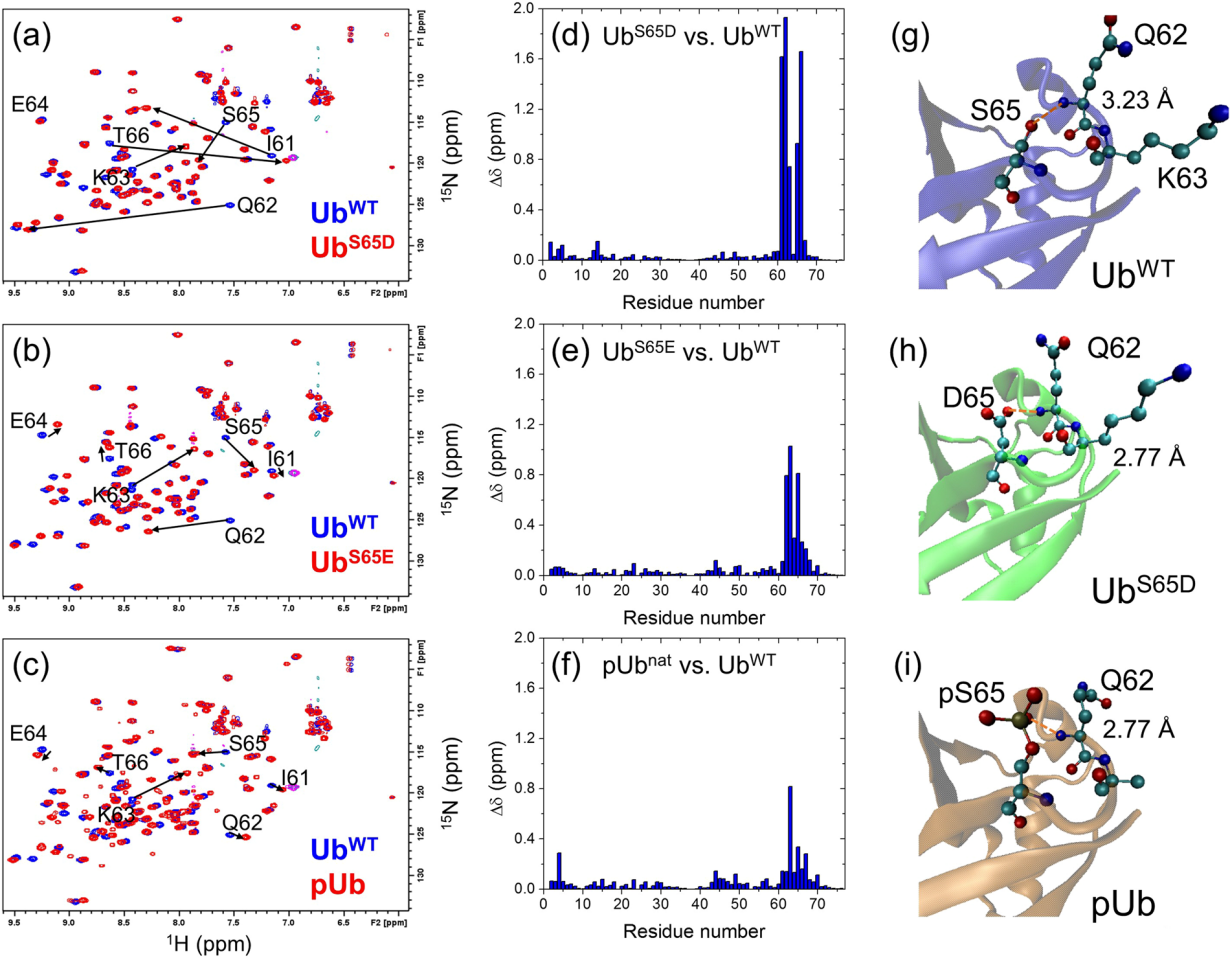
NMR and structural comparison of pUb and phosphomimic Ub variants with Ub^WT^. (**a**–**c**) Overlay of ^1^H-^15^N correlation spectra of (**a**) Ub^S65D^, (**b**) Ub^S65E^, and (**c**) pUb (all colored red) with the spectrum of Ub^WT^ (blue). Signals belonging to several residues around the modification site are indicated, their shifts are marked with arrows. (**d** and **e**) Spectral differences seen in panels (**a**–**c**) quantified as amide chemical shift perturbations in (**d**) Ub^S65D^, (**e**) Ub^S65E^, and (**f**) pUb versus Ub^WT^ as a function of residue number. (**g**–**i**) Comparison of the crystal structures of (**g**) Ub^WT^ (PDB ID: 1UBQ), (**h**) Ub^S65D^ (this work), and (i) pUb^nat^ (PDB ID: 4WZP); zoom on the area containing residue 65. The numbers indicate the distance (shown by the orange dashed line) between the backbone amide nitrogen of Q62 and the closest to it oxygen of the side chain of S65, D65, or pS65, respectively.

From the pH titration of pUb it became apparent that the (−2) charge of the phosphate group is necessary for the pUb^alt^ conformer to be present. This led us to hypothesize that the phosphomimetic Ub variants (Ub^S65D^ and Ub^S65E^) commonly used to mimic phosphorylation *in vivo* can only model the pUb^nat^ conformer, owing to the fact that neither an aspartate nor glutamate residue is able to attain a (−2) charge. To test the ability of the phosphomimics to structurally model both conformers, and to test the effects of a single negative charge at S65 on Ub structure, we collected ^1^H-^15^N NMR spectra for both Ub^S65D^ and Ub^S65E^. Surprisingly, both mutations resulted in very striking spectral perturbations (Figs 2a,b,d,e and S3e,f). However, the absence of any new signals in either phosphomimetic variant, in agreement with the previous observations^27^, indicates that no pUb^alt^–like conformer exists for Ub^S65D^ or Ub^S65E^. Puzzlingly, of the three S65 modifications considered here, S65D resulted in much greater magnitudes of CSPs vs. Ub^WT^ (Fig. 2a–f). To understand whether the observed large CSPs were due to structural or charge-related effects, we determined the structure of Ub^S65D^ using X-ray crystallography.

The structure of Ub^S65D^ was resolved to 1.2 Å. The two-molecule unit cell is shown in Figure S3a. Indexing and refinement statistics are shown in Table S1. The Ub^S65D^ structure shows little overall deviation from Ub^WT^ (PDB ID: 1UBQ) (Figure S3b). With the flexible C-terminal tail included, the Ub^S65D^ and Ub^WT^ structures superimpose with a backbone RMSD of 0.56 Å for chain A of the two-chain asymmetric unit and 0.94 Å for chain B. Removing the flexible-tail residues R74, G75, and G76 decreases RMSD to 0.50 Å for chain A, and 0.69 Å for chain B. The backbone structures of Ub^S65D^ and Ub^WT^ are very similar, even in the loop containing S65 (Figs 2g,h and S3d). There are minor positional differences in the side chains of residues 62 to 65. Elsewhere in the molecule, the greatest structural deviation is in the C-terminal tail, which is likely a result of the tail’s flexibility, as well as in the β1/β2 loop comprising residues T7 to G10. An examination of the polar contacts between Q62 and S/D65 showed noticeable differences in the backbone environment of Q62 in the mutant compared to Ub^WT^. The side chain oxygen (O_δ2_) of D65 in Ub^S65D^ is 0.44–0.47 Å closer to the backbone amide nitrogen of Q62 than the S65 oxygen in Ub^WT^ (Fig. 2g,h). At the same time, it is 0.94–0.95 Å farther from the backbone carbonyl oxygen of Q62.

Ub^S65D^ is also structurally similar to the crystallized pUb^nat^ conformer of pUb (PDB ID: 4WZP, Figs 2i and S3d)^27^. The backbone RMSD is 0.63 Å (chain A, tail residues not included). In the pUb structure, the closest phosphate oxygen to the amide group of Q62 is at about the same distance as the D65 side chain oxygen in Ub^S65D^ (Fig. 2i). Furthermore, for all three Ub variants considered here (Ub^WT^, Ub^S65D^, and pUb^nat^) the crystal structures predict a polar/hydrogen-bond contact between the side chain oxygen of residue 65 and the backbone amide nitrogen of Q62, as well as a hydrogen bond between the backbone amide nitrogen of residue 65 and the carbonyl oxygen of Q62. However, the chemical shift differences between Ub^S65D^ and pUb are nonetheless very striking (Figure S3f), suggesting that the phosphomimic does not fully reproduce the chemical environment of the pUb^nat^ conformer in solution.

### The effect of S65 phosphorylation on the ligand-binding properties of ubiquitin

**Figure 3:**
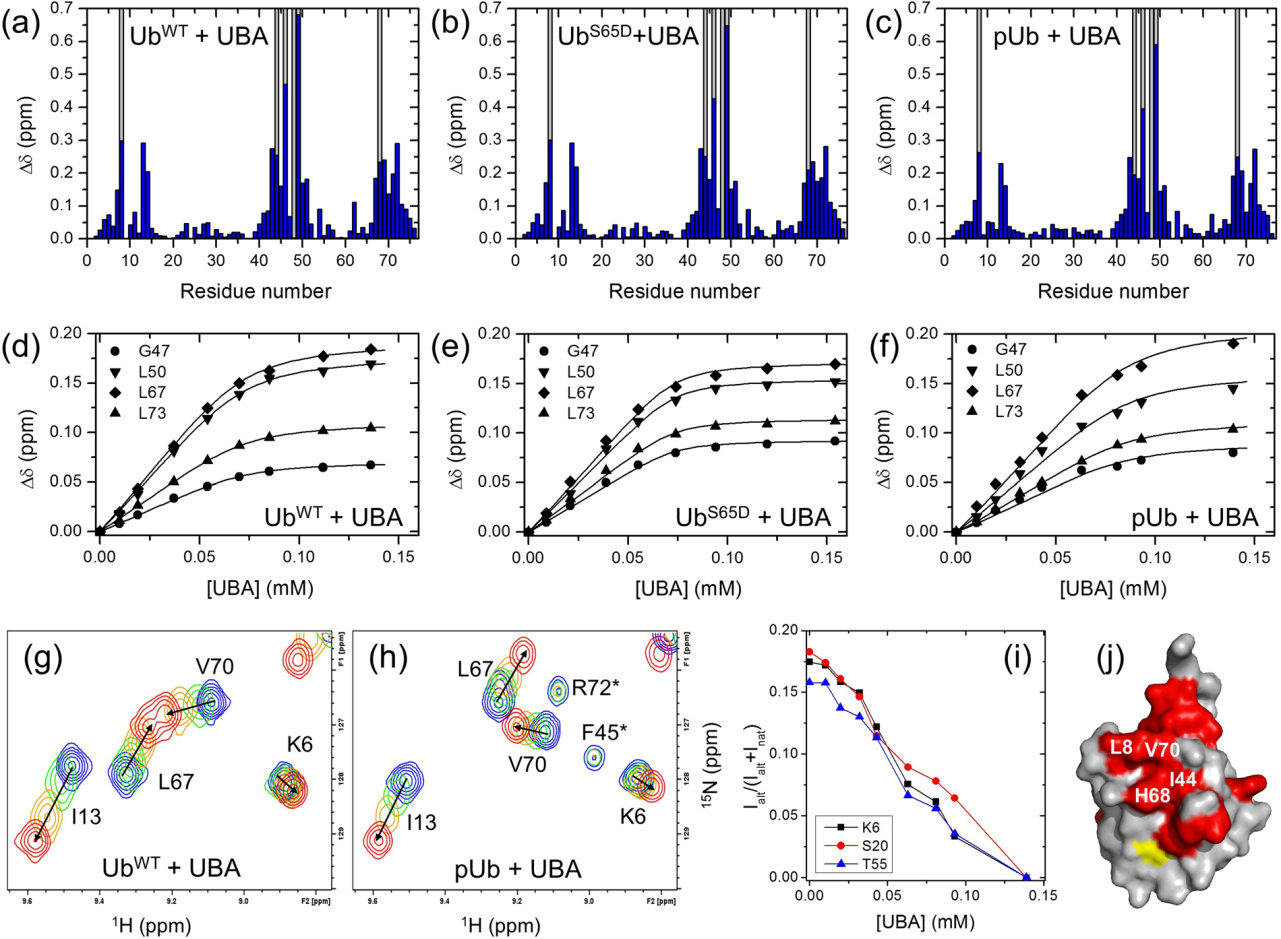
NMR titration analysis of binding of the UBA domain of ubiquilin-1 to the Ub variants studied here. (**a**–**c**) Spectral perturbations at the titration endpoint for each residue and (**d**–**f**) the representative titration curves for Ub^WT^, Ub^S65D^, and pUb. Blue bars in (**a**–**c**) show the magnitude of amide CSPs as a function of residue number in Ub, while the grey bars indicate residues exhibiting strong signal attenuations during titration (>75%). The curves in (**d**–**f**) correspond to a global fit of all analyzed residues to a single-site binding model (see Table S2**)**. (**g**,**h**) Fragments of the ^1^H-^15^N correlation spectra of Ub^WT^ (**g**) and pUb (**h**) illustrating NMR signal behavior in the course of UBA titration, colored from blue (free Ub or pUb) to red (titration endpoint). (**i**) The reduction in the relative intensity of the pUb^alt^ signals upon addition of UBA. (**j**) Map on the surface of Ub of the consensus residues (painted red) exhibiting strong CSPs and/or signal attenuations upon UBA binding. Hydrophobic patch residues are indicated. S65 is painted yellow.

We next tested the ability of pUb to bind to a well-characterized Ub-binding partner and one of the strongest Ub-binding domains^37^, the ubiquitin-associated domain (UBA) of human ubiquilin−1/hPLIC1^38^ involved in regulation of Ub-mediated proteasomal degradation of proteins^39^. We hypothesized that the structural perturbations in pUb due to the pUb^alt^ conformer would impair UBA binding. To determine binding affinity, we titrated ^15^N-labeled pUb with unlabeled UBA and monitored changes in the chemical shifts of the pUb signals. By monitoring the pUb^nat^ signals we found that pUb binds UBA with a comparable affinity to WT Ub (Fig. 3a–f, Table S2). By contrast, the pUb^alt^ signals barely shifted upon the addition of UBA (Fig. 3g and h) but instead uniformly decreased in intensity, to an even greater extent than upon reduction of pH (Figs 3i and S4). This suggests that the pUb^alt^ conformer is not involved in UBA binding, and the observed disappearance of the pUb^alt^ signals reflects a shift in the conformational equilibrium between pUb^alt^ and pUb^nat^ upon UBA binding to the latter. We also characterized the ability of the phosphomimetic Ub^S65D^ to bind the UBA, in order to test how well Ub^S65D^ can mimic pUb functionally. We found that Ub^S65D^ has a somewhat enhanced affinity for UBA compared to pUb and even Ub^WT^: the derived dissociation constant values were K_d_ = 4.9 ± 1.2 μM (Ub^WT^), 1.8 ± 0.5 μM (Ub^S65D^), and 8.0 ± 2.5 μM (pUb^nat^) (Fig. 3d–f, Table S2). For all three Ub variants, Ub^WT^, Ub^S65D^, and pUb, strong signal attenuations were observed for residues L8, I44, A46, K48, Q49, and H68 (Fig. 3a–c), indicating an intermediate or slow exchange on the NMR chemical shift timescale, as reported previously^37,38^. This suggests that the canonical ligand-binding interface remains intact in the pUb^nat^ conformer of the phosphorylated Ub (Fig. 3j).

We also examined the effect of S65 phosphorylation on Ub binding to K48-specific deubiquitinase OTUB1, which interacts with the hydrophobic patch surface on Ub or Ub_2_^40^. The addition of OTUB1 to ^15^N-labeled pUb resulted in significant attenuation of pUb^nat^ signals (Figure S5c), consistent with the expected 5–6 fold increase in the molecular mass upon pUb binding to a 31 kDa protein. The residue-specific shifts and attenuations of pUb^nat^ NMR signals map the OTUB1-interaction surface to the hydrophobic patch surface of pUb (Figure S5a,c,e). These results are consistent with our previous observations for OTUB1 binding to unphosphorylated Ub and Ub_2_^40^ and with the structural data indicating that S65 is not directly involved in interactions with OTUB1^41,42^. By contrast, only minimal shifts and signal attenuations were detected for pUb^alt^, suggesting that this conformer does not interact with OTUB1. The inability of pUb^alt^ to bind OTUB1 is in line with the observed inhibitory effect of S65 phosphorylation on polyUb disassembly by this enzyme^27^.

### Phosphorylation of S65 perturbs polyubiquitin conformations

**Figure 4:**
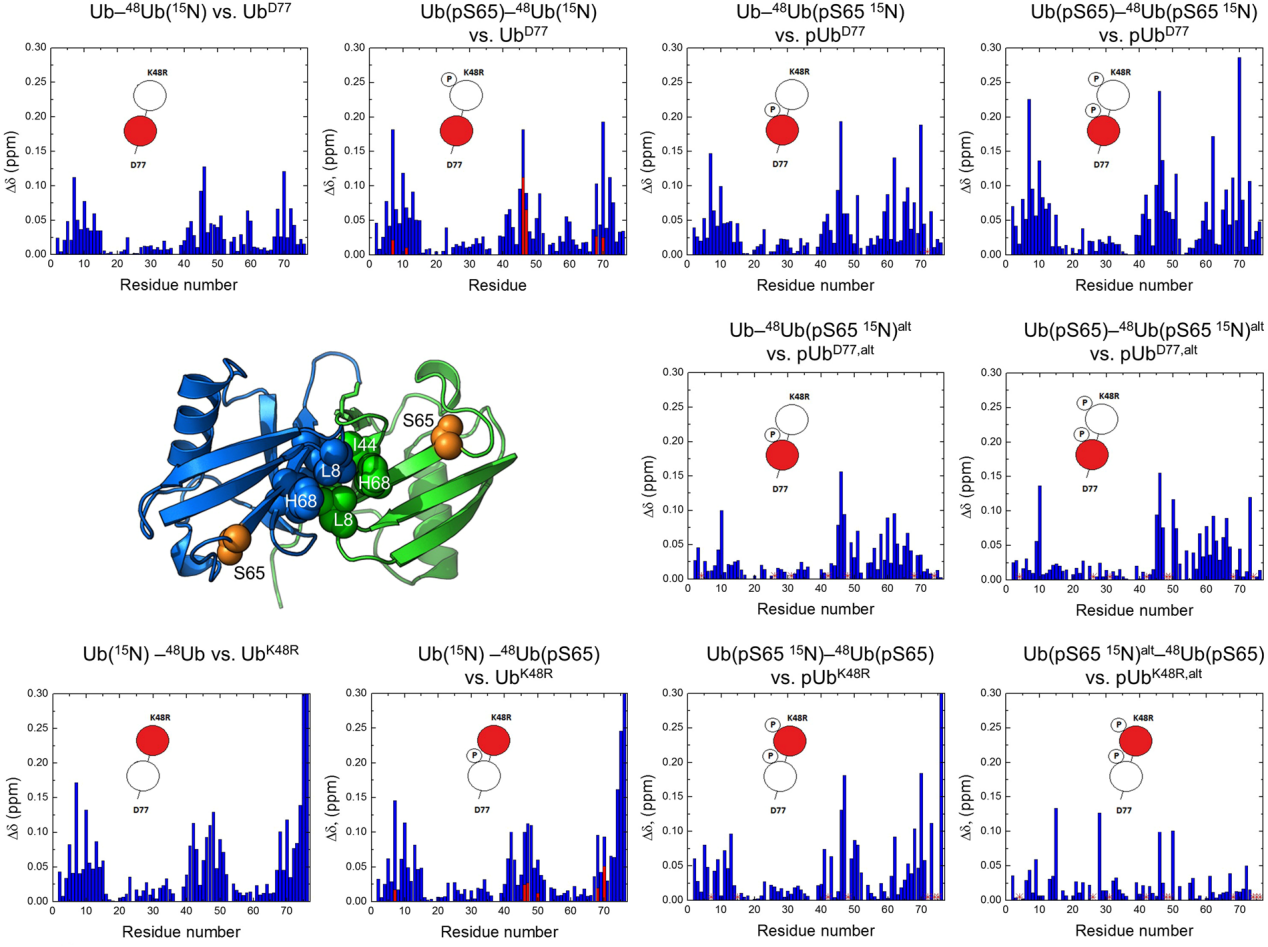
NMR analysis of the effect of Ub phosphorylation on the spectra of K48-linked Ub dimer. Each CSP plot shown here compares the spectra of a given Ub unit in the dimer with the respective monomeric Ub variant. Also shown are cartoon representations of each dimer, where phosphorylated Ub unit is marked with a “P” and the ^15^N-labeled unit (which acts as a reporter in each case) is colored red. Following the established nomenclature in the Ub field^44^, the Ub unit containing free C terminus is called “proximal” while the other Ub, conjugated to it through its C-terminal G76, is called “distal”. The proximal Ub has D77 as the C-terminal residue while the distal Ub contains a K48R mutation. The red bars on the plots for Ub(^15^N)–^48^Ub(pS65) and Ub(pS65)–^48^Ub(^15^N) indicate CSPs for the additional signals. Red asterisks indicate signals that could not be confidently assigned due to signal overlap. Also shown is the 3-D structure of the closed state of K48-linked Ub dimer (PDB ID: 1AAR); the hydrophobic patch residues L8, I44, V70 as well as H68 of the distal (blue) and proximal (green) Ubs are shown as spheres, the site of phosphorylation, S65, on each Ub is shown as orange spheres.

Given that phosphorylated polyUb chains are found in cells^28,29^ and PINK1 has been identified as a polyUb kinase^27^, understanding how S65 phosphorylation affects interdomain interactions and conformational properties of polyUb chains is critical to a complete understanding of the consequences of this modification. Since the pUb^alt^ conformer turned out to be binding incompetent with respect to an intermolecular interaction, we next examined how it would behave in an intramolecular setting, when the binding partner is another Ub within the same chain. Because linkage via K48 is the most abundant in cells^43^, and K48-linked Ub chains act as signals for proteasomal degradation^44^, we examined the effect of S65 phosphorylation on K48-linked Ub dimers. Specifically, we tested whether the Ub:Ub interface, a hallmark of the K48-linked Ub dimer^44–46^, is affected by the phosphorylation. For this we synthesized K48-linked dimers with S65 phosphorylated in one or both Ub units. Figure 4 contains schematic diagrams of every phosphorylated Ub dimer that was synthesized. Two versions of each dimer were made: one with ^15^N-labeled proximal Ub, and one with ^15^N-labeled distal Ub (the labeled unit is shown as a red circle in Fig. 4). To denote these dimers, we will use the Ub chain nomenclature introduced by Nakasone *et al*.^47^, in which Ub units are written in the distal to proximal direction (left to right). We append this nomenclature by the addition of a (pS65) to indicate the phosphorylated Ub. For example, Ub(^15^N)–^48^Ub(pS65) denotes a K48-linked dimer ^15^N-labeled on the distal Ub and phosphorylated on the proximal Ub. For brevity, we will omit the K48R and D77 designations in our notations, unless necessary, as all our Ub dimers possess these mutations in the distal and proximal units, respectively.

The first two Ub dimers we tested contained a single phosphate group, and were ^15^N-labeled on the unphosphorylated Ub unit: Ub(^15^N)–^48^Ub(pS65) and Ub(pS65)–^48^Ub(^15^N). For both dimers, the CSP patterns at pH 6.8 resemble those in the corresponding unphosphorylated dimers (Fig. 4), indicating the formation of the Ub:Ub interface^45^. The CSPs for the residues forming the interface were somewhat stronger in the proximal Ub and weaker in the distal Ub compared to those in the unphosphorylated dimers (see also Figures S6c and S7c). In both cases the amide signals shifted in the same direction as in the unphosphorylated dimer (Figures S6d and S7d), suggesting similar interatomic contacts. Furthermore, only negligible CSPs were observed at pH 4.6, indicating absence of the Ub:Ub interface at low pH (Figure S8). This behavior is in full agreement with the pH-dependent conformational switch reported for the unphosphorylated dimer^45,48^.

To our surprise, several residues in the ^15^N-labeled Ub units exhibited new signals resulting from phosphorylation of the opposite Ub unit in the dimer, indicating that these residues sense two different environments. We denote these as “additional” signals. The NMR spectra allowed us to rule out contamination of our samples with either unreacted Ub monomer or unphosphorylated dimer (Figures S6a,b and S7a,b), thus confirming that the new signals are genuine to the phosphorylated dimer. Interestingly, the residues exhibiting these additional signals cluster around the Ub:Ub interface (Figures S6f and S7f). We noted that these signals were much closer to the signals from monomeric Ub than to Ub dimer, and in many cases we observed a linear movement, from the monomer signal to the additional signal to the dimer signal (Figures S6e and S7e). We therefore hypothesize that the additional signals correspond to those dimers where the phosphorylated Ub unit is in the pUb^alt^ state. Judging by the smaller signal shifts, these dimers have a weaker interface. In line with our hypothesis, the additional signals were absent at pH 4.6, where pUb^alt^ is only weakly populated (Fig. 1d), although this could also reflect the disappearance of the Ub:Ub interface at low pH^45,48^. Overall, we conclude that while the Ub:Ub interface is generally preserved in the singly phosphorylated Ub dimers, even a single phosphate group on one Ub unit is enough to perturb the conformational states of the K48-linked Ub dimer.

We next examined three additional dimers: Ub–^48^Ub(pS65, ^15^N), Ub(pS65)–^48^Ub(pS65, ^15^N), and Ub(pS65, ^15^N)–^48^Ub(pS65). The latter two are phosphorylated on both Ub units. For each dimer, CSPs were determined for both pUb^nat^ and pUb^alt^ conformers (Fig. 4). Our findings can be summarized as follows. First, despite the relatively tight^45,46^ interface between the two Ub units, the pUb^alt^ conformer was still present in both the proximal and distal units. Second, the largest CSPs for each phosphorylated dimer were found in the same residues as for the unphosphorylated dimers, indicating that the Ub:Ub interface is generally preserved even when both Ubs are phosphorylated. Third, the magnitude of the CSPs in the proximal Ub appears to increase with each additional phosphate (Fig. 4) suggesting strengthening of the interface. Fourth, the signal shifts for the pUb^alt^ conformer upon dimer formation were overall significantly smaller than those for the pUb^nat^ conformer, suggesting that the pUb^alt^ conformer does not participate in the Ub:Ub interface or at least plays a lesser role in forming it (Fig. 4). Lastly, when using the Ub^D77^ mutant (phosphorylated at S65) as a reference for the proximal Ub in the dimers, we observed that the additional Asp (D77) at Ub C terminus slightly stabilized the Ub^alt^ conformer (Figure S9a), possibly through electrostatic interactions of the additional negative charge on the C terminus with the positively charged residues surrounding the β5 strand.

## Discussion

Our study confirmed that the alternate NMR signals (pUb^alt^) caused by phosphorylation of S65 are not an artifact of Ub:Ub binding but in fact reflect the internal conformational equilibrium between the native-like and an alternate states of pUb. Consistent with the previous report^27^, phosphomimetic mutations do not result in the novel conformation of pUb. Furthermore, they do not mimic the chemical shifts observed for pUb, even for the pUb^nat^ conformer (Figure S3f). This raises the question: what differences between Ub^S65D/E^ and pUb are responsible for the presence of the pUb^alt^ conformer? The dramatic reduction of the pUb^alt^ conformer signals at low pH provides a clue, suggesting that the protonation state of the phosphate group regulates the equilibrium between the two pUb conformers. At pH 4.6, the phosphate group is predominantly singly deprotonated, carrying the (−1) charge. As the pH increases toward the pK_a2_ of phosphate, the fraction of the doubly-deprotonated phosphate groups, with the (−2) charge, increases, and so does the fraction of pUb^alt^. Thus the presence of this conformer appears to be coupled to the protonation/charge state of the phosphate on S65.

**Figure 5:**
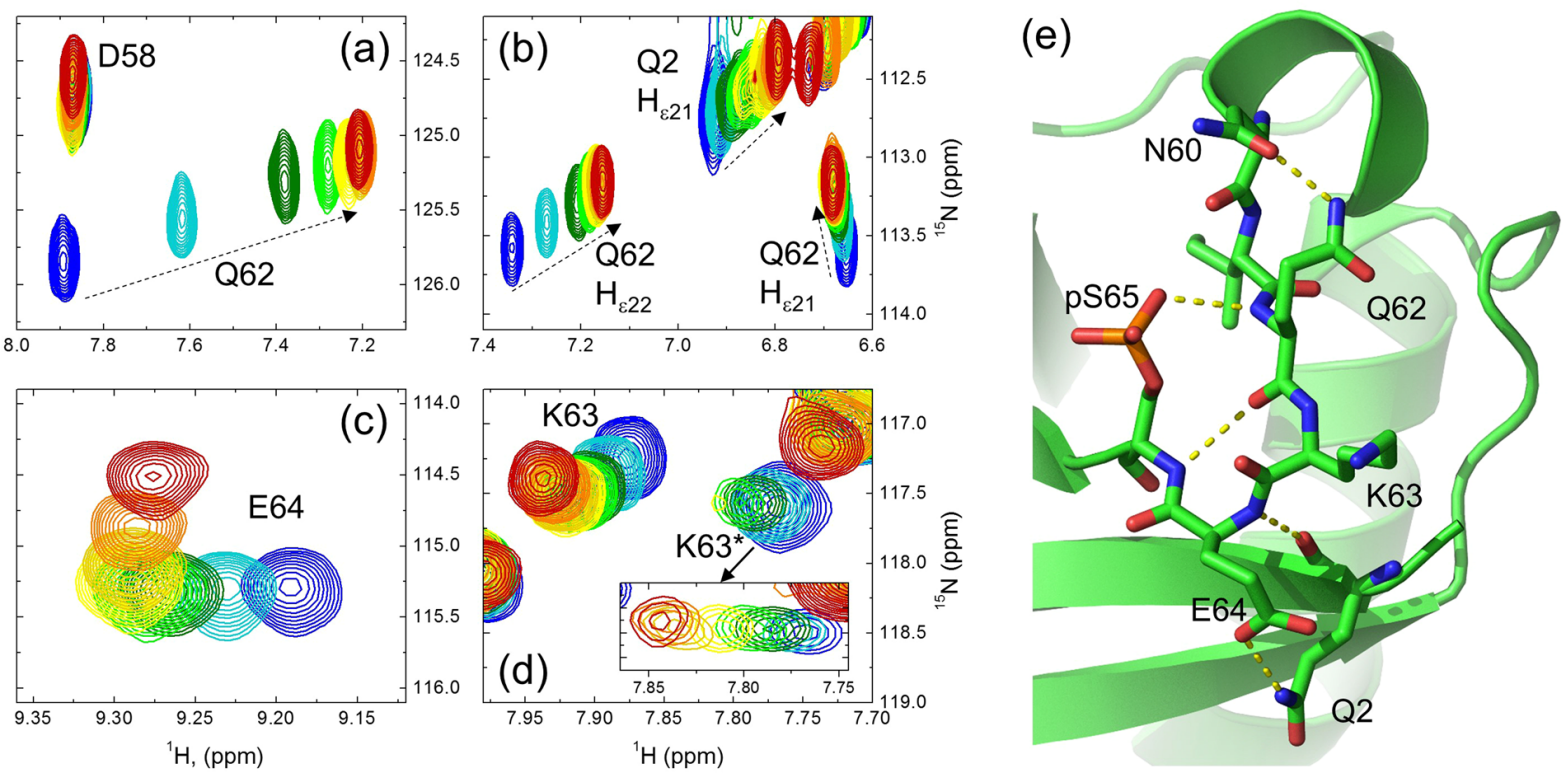
Differential behavior of NMR signals with pH relates to the hydrogen bonding network around the phosphorylation site. (**a**–**d**) Shifts of ^1^H-^15^N NMR signals of the backbone amides of (**a**) Q62, (**c**) E64, (**d**) K63, and (**b**) the side chain amides of Q62 and Q2 upon gradual change in pH from 7.6 (blue) to 4.8 (red). Both pUb^nat^ and pUb^alt^ signals of K63 are seen in (**d**). The inset depicts the movement of the K63 pUb^alt^ signal (K63*); the intensities were artificially scaled up as the pH decreased in order to follow the vanishing signal. To guide the eye, the dashed arrows in (**a** and **b**) are drawn in the direction of signal shifts as the pH decreases. (**e**) Fragment of the crystal structure of pUb^nat^ (PDB ID: 4WZP) showing the relevant residues (in stick representation) surrounding the phosphorylation site and the PyMol-predicted polar/hydrogen bonds (yellow dots) between them.

This in turn raises an intriguing question regarding the structural mechanism that relates protonation/deprotonation of pS65, expected to be ultrafast for a solvent-exposed phosphate group (e.g.^49,50^), to the slow (ca. 2 per sec) exchange between the pUb^nat^ and pUb^alt^ conformers. One can speculate that hydrogen bonds between the phosphate group and surrounding atoms in Ub might slow down the proton transfer between the phosphate and water. In fact, the crystal structure of pUb^nat^ suggests a hydrogen bond between a phosphate oxygen of pS65 and the backbone amide nitrogen (N_H_) of Q62, and indeed, our NMR study revealed a strong pH-dependent shift (0.66 ppm in ^1^H and 0.65 ppm in ^15^N) of the Q62 amide signal in pUb but not in Ub^WT^ (see Figs 5a and S2a,b). Interestingly, we also found that the two side-chain amide hydrogens of Q62 in pUb behave differently with pH. Specifically, the signal of the H_ε22_ proton showed a dramatic shift (0.19 ppm) over the course of our pH titration, while the H_ε21_ signal shifted much less (see Fig. 5b). Both signals were virtually stationary in the pH titration of Ub^WT^ (Figure S2b), indicating that the shifts detected in pUb arise from titration of the phosphate. The differential behavior of the NMR signals of the two hydrogens suggests a specific hydrogen-bonding pattern involving Q62. Indeed, the crystal structure of pUb^nat^ suggests a hydrogen bond between the side chain N_ε2_ atom of Q62 and the side chain O_δ1_ oxygen of N60, mediated by one of the amide protons (likely H_ε22_) of Q62 (Fig. 5e). Of note, this hydrogen bond is not present in Ub^WT^. Together with the hydrogen bonds between the phosphate group and N_H_ of Q62, between N_H_ of S65 and C’O of Q62, as well as between the side chain oxygen of E64 and the N_ε2_ nitrogen of Q2 (not present in Ub^WT^ or Ub^S65D^), these interactions form a network of hydrogen bonds involving pS65 (Fig. 5e). This suggests an intriguing possibility, yet to be tested, that these interactions might play a role in slowing down and relaying the protonation/deprotonation of the pS65 phosphate group to the transitions between the pUb^nat^ and pUb^alt^ conformers.

Interestingly, the pUb^alt^ conformer did not completely disappear at low pH but plateaued at ca. 10% of the total protein (Fig. 1d). This suggests that other factors contribute to its stabilization at low pH. In this regard we noticed that pUb^nat^ amide signals of the residues close to pS65 (most prominently, K63 and E64) did not shift linearly with pH but instead changed the direction of the shift below pH 5.6 (Fig. 5c and d). This could reflect protonation of the neighboring ionizable groups (e.g., E64, H68) and/or some local structural rearrangement in pUb^nat^. Note that such behavior is not present in the Ub^WT^ spectra.

Given the role of the protonation state of the phosphate in controlling the conformational equilibrium of pUb, the inability of aspartate and glutamate to adopt a (−2) charge on their side chain explains the observed structural and spectroscopic differences between phosphomimetic and phosphorylated Ub and suggests that phosphomimetic Ub is a poor structural model for pUb. It should nonetheless be noted that even a (−1) charge on residue 65 in Ub is sufficient to cause large CSPs (Fig. 2d and e), indicating that the charge at this position significantly affects the chemical environment of the backbone amides of the neighboring residues.

Many studies have used such phosphomimetic mutations to simulate phosphorylation of parkin^17,18,29,51^ and mono-^17,18,51,52^ and polyUb^29^. While these variants have generally been sufficient to model Ub phosphorylation in several cases, differences between Ub^S65D/E^ and pUb have been reported^27,53^, matching our observations. Furthermore, although Ub^S65D/E^ mutants have proven to be adequate surrogates for S65 pUb in its role as a parkin activator^17,18,29^, it is unclear whether these phosphomimics will be adequate in studies of other potential roles of pUb, particularly those that involve the pUb^alt^ conformer, as well as for other phosphorylation sites on Ub.

The canonical ligand-binding surface of Ub does not appear to be disrupted either by phosphorylation (in case of the pUb^nat^ conformer) or by phosphomimetic mutations of S65, as demonstrated by our experiments with the UBA of ubiquilin-1. However, our detailed analysis of the behavior of the NMR signals of the two pUb conformers upon addition of UBA suggests that pUb^alt^ is incapable of binding to a ligand like ubiquilin-1 UBA, and it is the pUb^nat^ conformer that binds the UBA. Similar conclusions can be drawn from our analysis of OTUB1 binding to pUb. From a structural perspective, the β5-strand slippage by two residues in pUb^alt^ proposed in^27^ and recently shown by NMR^36^ disrupts the canonical Ub hydrophobic surface patch by shifting V70 to where H68 is in Ub^WT^ and by placing a positively charged side chain of R72 at the location of V70 in Ub^WT^ (Figure S10). Overall, such a shift would amount to mutating V70 to R, H68 to V, and possibly T66 to H. From this point of view the inability of pUb^alt^ to bind UBA is in line with the fact that the V70R mutation in Ub is not tolerated by yeast cells^54^. This also suggests that the pUb^alt^ conformer might be incapable of binding to other Ub-receptors that target the L8-I44-V70 hydrophobic patch of Ub, which could potentially impact pUb recognition. The weak “additional” signals detected here in K48-linked Ub dimers and reflecting a weaker interface between Ub and pUb^alt^ seem to support these predictions. Our data indicate that the presence of the pUb^alt^ conformer did not significantly impact the overall strength of UBA binding, likely due to the dynamic equilibrium between pUb^alt^ and pUb^nat^. Indeed, our binding assays show that UBA binding to pUb stabilizes the pUb^nat^ conformer. Published structures of pUb in complex with parkin^55,56^ do not show any evidence of the pUb^alt^ conformer. However, although the latter conformer has not yet been found to be relevant for parkin activation, it may perhaps be relevant for other interactions. It remains to be seen whether a ligand exists that can specifically recognize and stabilize the pUb^alt^ conformer of pUb as suggested recently^57^.

Our Ub dimer studies also provide insights into the impact of phosphorylation on the structure of Ub chains. K48-linked Ub dimers exist in solution in a dynamic equilibrium of multiple conformational states, including the “closed” state (wherein the hydrophobic patch residues form the “canonical” noncovalent Ub:Ub interface) and various “open” states, some of which are pre-organized for receptor recognition^48^. The CSPs (dimer vs. monoUb) reflect this equilibrium^45^ and therefore are uniquely suited for monitoring changes in the conformational ensemble of K48-linked Ub dimers. We found that the Ub:Ub interface characteristic of K48-linked chains at neutral pH is generally preserved upon phosphorylation of S65 and disappears at low pH (Figure S8), as in the unphosporylated dimer^45^. However, the additional NMR signals detected in the unphosphorylated Ub units of K48-linked dimers show that the conformational changes in the phosphorylated Ub unit are acutely sensed by the opposite Ub unit in the chain. The smaller magnitude of the CSPs (Fig. 4) associated with these signals suggests a weaker interface, i.e. a less populated closed state compared to the unphosphorylated K48-linked dimer. We hypothesize that this reflects those dimers where the phosphorylated Ub unit is in the pUb^alt^ conformer. Moreover, for the dimers phosphorylated on both Ub units we did observe an additional (third) signal for G47 (Figure S9b). Taken together, our results indicate that, through the pUb^alt^ state, Ub phosphorylation at S65 affects not only Ub-receptor interactions, but also the conformations that K48-linked Ub chains adopt in solution, and might serve as an additional mechanism of regulation in polyUb-mediated signaling pathways.

Ub phosphorylation at S65 presents and interesting and potentially important example that not only the negative sign but also the actual value of the charge/protonation state of a phosphorylated side chain can result in different structural and recognition properties of a protein. This can have implications for phosphorylation of other proteins. While the biological role of the pUb^alt^ conformer of pUb remains elusive, it is clear, however, that a phosphomimetic mutation is not a foolproof model for phosphorylated protein, and using phosphomimics to study the effects of phosphorylation *in vitro* and *in vivo* should be done with care.

## Materials and Methods

Ub^WT^ and both phosphomimetic variants (Ub^S65D^, Ub^S65E^) were grown and purified using the standard Ub purification protocol^45,58^. Ub used for NMR experiments was grown in ^15^N-enriched minimal media. PINK1 was grown and purified as described previously^19^, although Rosetta cells were used in place of BL21-RP. The UBA domain of human ubiquilin-1 was expressed and purified as described^38^. The UBA construct used in this study contained a V545Y mutation in order to enable its concentration determination through absorbance at 280 nm. Human OTUB1 was expressed and purified as described^40^.

The protocol for Ub phosphorylation was adapted from^19,27^. To generate pUb, 2 mM Ub were incubated with 32 μM maltose-binding protein-tagged-*Tc*PINK1 in a 0.30 mL volume. The reaction buffer contained 50 mM Tris-HCl (pH 7.5), 10 mM MgCl_2_, and 2 mM DTT. 10 mM ATP was added to start the reaction. The reaction was incubated at room temperature for 3 hours. ESI-MS was used to confirm phosphorylation of Ub (Figure S1b,c). Mass spectrometry was performed on a JEOL AccuTOF-CS mass spectrometer in positive electrospray ionization mode.

The phosphorylated Ub was separated from unphosphorylated protein using a SPHP high performance cation exchange column (GE Healthcare). Column equilibration and wash buffer was 50 mM ammonium acetate (pH 4.5). Elution buffer was wash buffer plus 1 M NaCl. The phosphorylated protein eluted at 10% elution buffer. Purity was confirmed by ESI-MS and phosphorylation at the S65 site was confirmed by matching the NMR spectrum of the protein to published spectra^27^.

K48-linked Ub dimers were prepared enzymatically (see also refs^45,58^). First, the corresponding Ubs were phosphorylated as monomers and purified using the protocol detailed above. The formation of conjugates longer than dimers was blocked using chain terminating mutations, namely, a K48R mutation in the distal Ub and an addition of D77 at the C-terminus of the proximal Ub. Both Ub^K48R^ and Ub^D77^ monomers were mixed in a 1:1 ratio for the dimerization reaction. The reaction contained 2 mM total Ub, 20 μM E2-25K, 1 μM human E1, 2 mM ATP, 5 mM MgCl_2_, 10 mM creatine phosphate, 0.6 U/mL inorganic phosphate, 0.6 U/mL creatine phosphokinase, and 3 mM TCEP in 50 mM Tris, pH 8.0. Dimers were separated from unreacted monomers using high-performance cation chromatography followed by size exclusion chromatography.

Residues used for the analysis of pH titration of pUb were selected based on several criteria: (1) NMR signals that overlapped partially or entirely with each other were excluded. (2) Signals that could not be unambiguously assigned due to attenuation or migration were excluded. (3) Signals not present in the alternate conformer were excluded.

NMR experiments were performed on a Bruker Avance–III 600 MHz spectrometer equipped with the TCI cryoprobe. All experiments were performed at 23 °C except for temperature dependence studies. The buffer for all NMR samples (except for pH titration) contained 20 mM sodium phosphate, 0.02% NaN_3_ and 7% D_2_O, the pH was 6.8. Total correlation spectroscopy (TOCSY) and nuclear Overhauser effect spectroscopy (NOESY) were used for signal assignments of Ub^S65D^ and Ub^S65E^. NMR signal assignments for both pUb conformers were from reference^27^; we corrected assignments for G53 and G75. ^1^H-^15^N SOFAST-HMQC was used for most experiments, while HSQC was used for analysis of the temperature dependence. ^15^N relaxation experiments were performed as described^59^. NMR data were processed using TopSpin (Bruker Inc.) and analyzed using Sparky^60^ and in-house Matlab programs. Amide chemical shift perturbations (CSPs) for each residue were quantified as

$${\rm{\Delta }}{\delta }=\sqrt{{\rm{\Delta }}{{\delta }}_{H}^{2}+{({\rm{\Delta }}{{\delta }}_{N}/5)}^{2}},$$

where Δδ_H_ and Δδ_N_ are shifts of the ^1^H and ^15^N resonances, respectively. The fraction of pUb molecules in the pUb^alt^ conformation was determined as

$${{f}}_{{alt}}={{I}}_{{alt}}/({{I}}_{{nat}}+{{I}}_{{alt}}),$$

where *I*_*alt*_ and *I*_*nat*_ are signal intensities for a given residue corresponding to pUb^alt^ and pUb^nat^ conformations, respectively.

The thermodynamic parameters, Δ*H* and Δ*S*, for the equilibrium between the two pUb conformers were obtained using a linear regression analysis of the dependence of their relative populations,

1 $$\mathrm{ln}({{I}}_{{alt}}/{{I}}_{{nat}})=-\frac{{\rm{\Delta }}{G}}{{RT}}=-\frac{{\rm{\Delta }}{H}}{{RT}}+\frac{{\rm{\Delta }}{S}}{{R}},$$

on the reciprocal temperature, according to the Boltzmann distribution.

The pH dependence of the fraction of pUb molecules in the pUb^alt^ conformation was fit to the following equation reflecting the acid-base equilibrium:

2 $$\frac{{{I}}_{{alt}}}{{{I}}_{{alt}}+{{I}}_{{nat}}}={offset}+{scale}\times {{\rm{10}}}^{{pH}-{pKa}}.$$

The shifts in NMR signals of Ub and pUb^nat^ as a function of pH were quantified as CSPs, and the normalized CSPs were fit to the following equation:

3 $${\rm{\Delta }}{{\delta }}_{{normalized}}\equiv \frac{{\rm{\Delta }}{\delta }({pH})}{{\rm{\Delta }}{\delta }({pH}7.6)}={offset}+{\rm{\Delta }}{{\delta }}_{{\rm{\max }}}\times {10}^{{pH}-{pKa}},$$

where Δδ(pH) for each residue was computed as the CSP at a given pH value versus the signal position at pH 4.8.

The dissociation constant, *K*_d_, for UBA binding was obtained by fitting the measured CSP values (Δ*δ*) in Ub or Ub variants to a single-site binding model^61^:

4 $${\rm{\Delta }}{\delta }={\rm{\Delta }}{{\delta }}_{\max }\{{[P]}_{t}+{[L]}_{t}+{K}_{d}-\sqrt{{({[P]}_{t}+{[L]}_{t}+{K}_{d})}^{2}-4{[P]}_{t}{[L]}_{t}}\}/(2{[P]}_{t}),$$

where Δ*δ*_max_ is the CSP at saturation and [*P*]_t_ and [*L*]_t_ are the total concentrations of the protein (Ub) and the ligand (UBA), respectively. *K*_d_ and Δ*δ*_max_ were treated as global fitting parameters using an in-house Matlab program Kdfit^61^.

Crystallization conditions for Ub^S65D^ were 0.1 M Tris pH 8.0, 0.2 M MgCl_2_, and 25% polyethylene glycol (PEG) 3350. Protein concentration was 4.23 mM and protein in 20 mM Hepes buffer was mixed 1:1 with crystallization buffer. Indexing of X-ray diffraction data for Ub^S65D^ was performed with Mosflm^62^. Phenix^63^ was used for molecular replacement. The crystal structure was refined using CCP4i^64^ and Coot^65^ software. Visual Molecular Dynamics^66^ was used for structural alignment of proteins, as well as for generation of figures.

### Data Availability

The atom coordinates of Ub^S65D^ have been deposited with the Protein Data Bank (accession code: PDB ID: 5W46).

The datasets generated during and/or analyzed during the current study are available from the corresponding author on reasonable request.

## Electronic supplementary material

Supplementary Information
