# Supplementary material for: Impact of different ionization states of phosphorylated Serine-65 on ubiquitin structure and interactions
Source: Sci Rep. 2018 Feb 8;8:2651. doi: 10.1038/s41598-018-20860-w (PMC5805711; doi:10.1038/s41598-018-20860-w)
Supplement: Supplementary file 1 — Supplementary Information [file 41598_2018_20860_MOESM1_ESM.pdf]

Supplementary Information

Impact of different ionization states of phosphorylated Serine-65 on ubiquitin structure and interactions

Yaniv Kazansky, Ming-Yih Lai, Rajesh K. Singh, David Fushman

Table S1. Indexing and refinement statistics for Ub^{S65D} crystal structure

**Data Collection**	
Wavelength (Å)	0.9792
Space Group	P1 2_1 1
Unit Cell Dimensions	
a, b, c (Å)	33.456 48.248 41.707
α, β, γ (°)	90.000 99.026 90.000
Resolution Range (Å)	48.25-1.18 (1.25-1.18)
R_{merge}	0.041 (0.285)
R_{meas}	0.051 (0.380)
I/σI	19.8 (2.8)
Completeness (%)	97.52 (87.5)
Multiplicity	4.6 (2.6)
**Refinement**	
Resolution Range (Å)	41.19-1.18
No. Reflections	39564
R_{free} Test Set	5.0
Reflections used for R_{free}	2097
R_{work}	0.1782
R_{free}	0.1899
Number of atoms	1318
Protein	1203
Waters	114
Ligand/ion	1
Wilson B-factor (Å^2)	11.284
RMS (bond lengths) (Å)	0.0099
RMS (angles) (°)	1.4484
Ramachandran favored (%)	99.30
Ramachandran allowed (%)	0.70
Ramachandran outliers (%)	0

Table S2. The results of NMR titration assays for UBA binding to Ub variants studied here. The K_d values reported here represent the mean and standard deviation of the individual K_d values obtained from fitting the titration data for the indicated residues separately, as well as the results of the global fit of the titration data for all these residues in the given Ub variant taken together.

Ub variant	Residues Used	K_d (μM) (mean ± std)	K_d (μM) (global fit)
Ub^{WT}	Q40 Q41 R42 G47 L50 D52 L56 Q62 L67 L73 R74 G75	4.9 ± 1.2	4.8
Ub^{S65D}	F4 K6 I23 Q40 Q41 G47 L50 I61 E64 T66 L67 L73 G75	1.8 ± 0.5	1.7
pUb	F4 K6 G10 Q40 Q41 G47 L50 D52 Q62 T66 L67 L73 R74 G75	8.0 ± 2.5	7.0

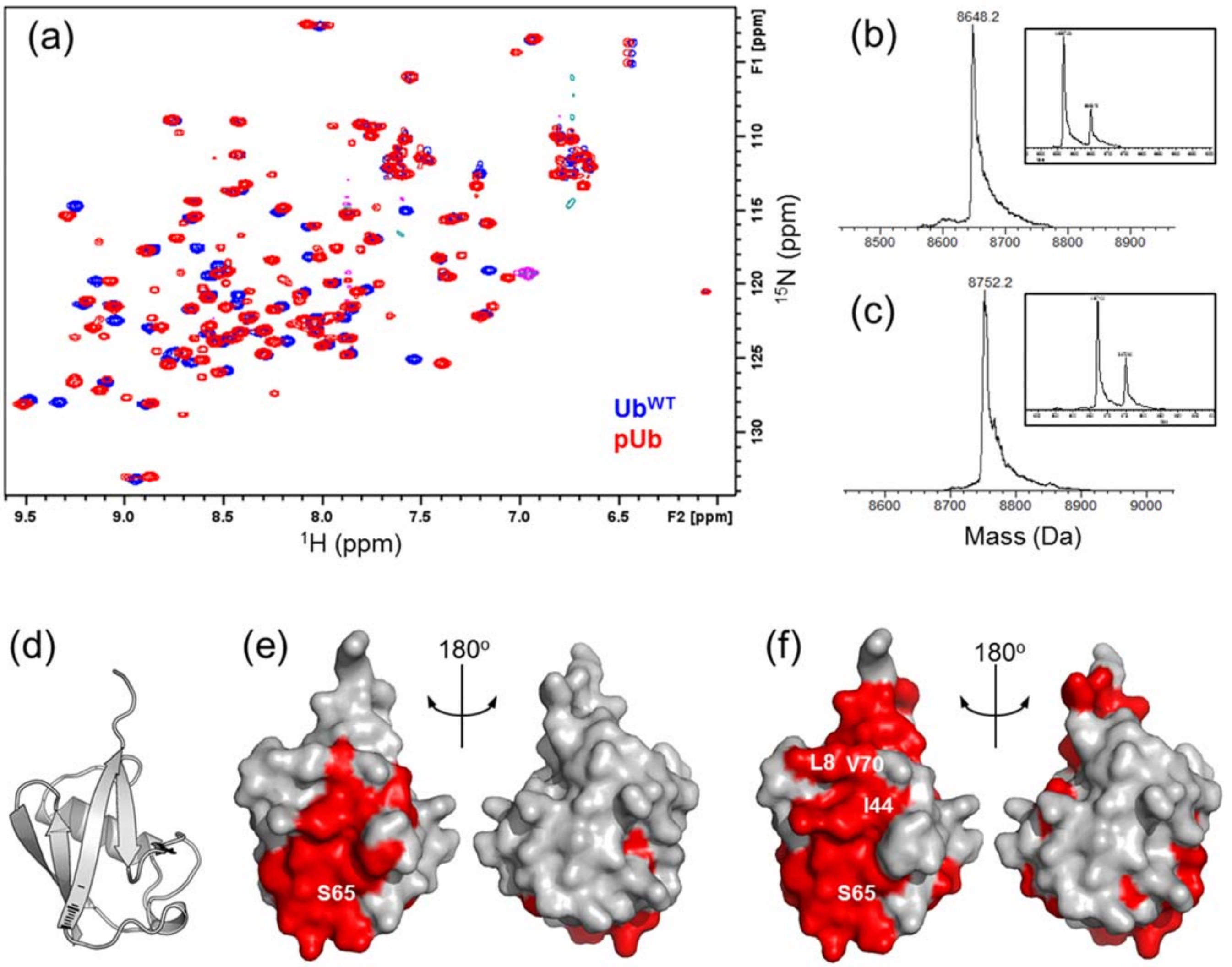

Figure S1. Preliminary characterization of pUb by NMR and mass spectrometry (MS). (a) Overlay of ^{1}H-^{15}N SOFAST-HMQC spectra of pUb (red) and Ub^{WT} (blue) at pH 6.8. (b-c) ESI-MS spectrograms of the post-purification pUb. Insets show post-kinase reaction mix of (b) unlabeled and (c) ^{15}N-labeled Ub. (d-f) Mapping the residues that exhibit strong amide chemical shift differences between Ub^{WT} and pUb. (d) Ribbon cartoon of Ub^{WT} structure (PDB ID 1UBQ). The molecule is oriented such that its hydrophobic patch surface faces the reader. (e) Residues with strong CSPs (> 0.076 ppm) for Ub^{nat} resonances are painted red on the surface of Ub. (f) Residues with strong CSPs (> 0.2 ppm) for Ub^{alt} resonances are painted red on the surface of Ub. The actual CSPs are shown in Figure S3f. In panels (e) and (f) the structure shown on the left has exactly same orientation as in panel (d), and the structure on the right is 180° rotated about the vertical axis. The locations of the hydrophobic patch residues V8, I44, V70, and of the phosphorylation site (S65) are indicated.

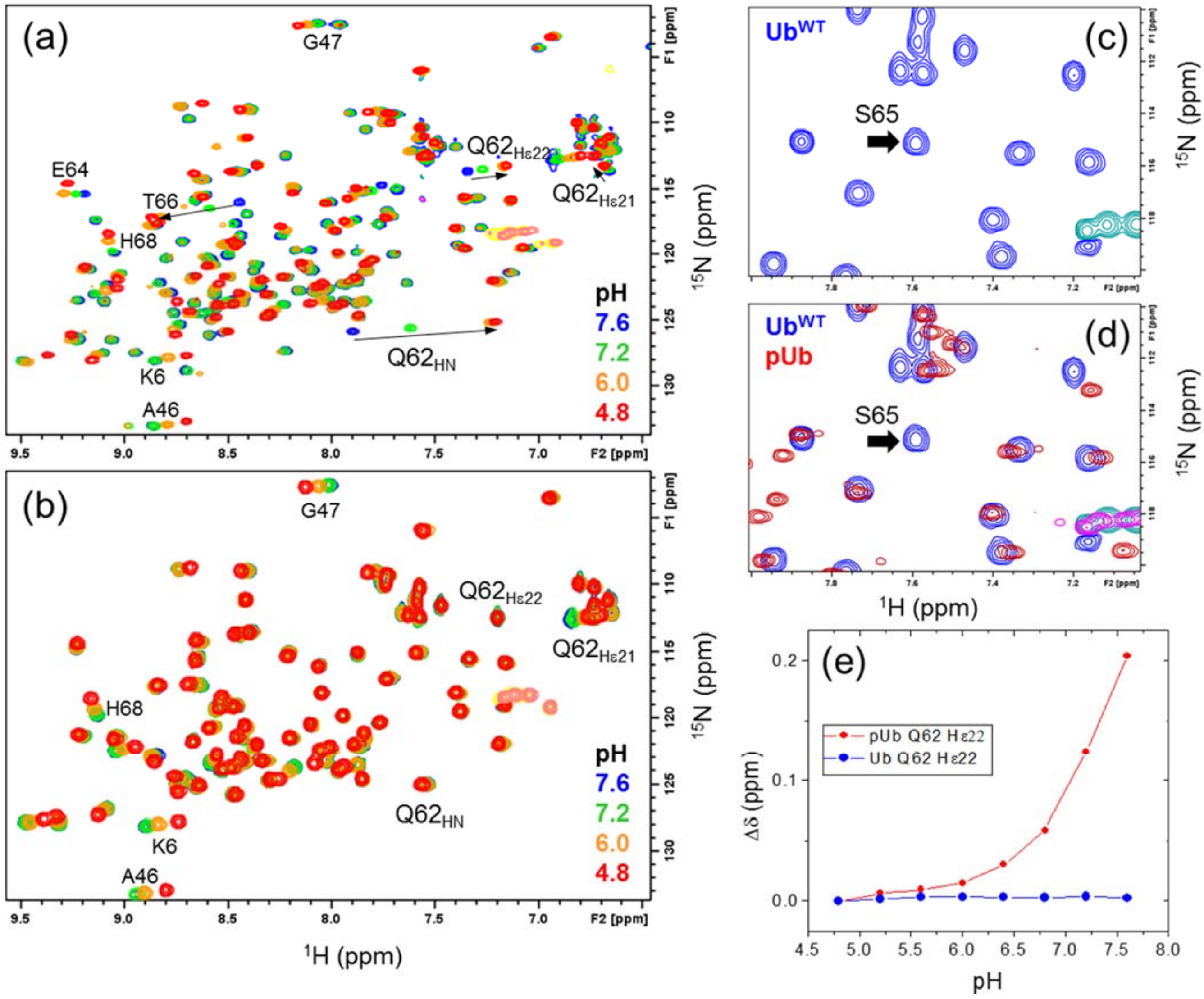

Figure S2. pH dependence of the NMR spectra of pUb. (a-b) Overlay of the ^{1}H-^{15}N SOFAST-HMQC spectra of (a) pUb and (b) Ub^{WT} recorded at various pH values (as indicated). The large signal shifts of the backbone and side chain amides of Q62 in pUb spectra are marked and indicated by arrows. This is in stark contrast with the behavior of these signals in Ub^{WT} where they show only minor shifts with pH. Also indicated are several residues that exhibit similar pH-dependent shifts in both pUb and Ub^{WT}. (c) Fragment of ^{1}H-^{15}N SOFAST-HMQC spectrum of Ub^{WT} at pH 4.8. The position of the S65 signal is marked. (d) Overlay of the ^{1}H-^{15}N SOFAST-HMQC spectra of pUb (red) and Ub^{WT} (blue, from panel (c)) at pH 4.8, the S65 signal is marked. The absence of the WT S65 signal in the pUb spectrum confirms that the phosphate is still attached to S65 at this pH. (e) Comparison of the pH-dependence of the CSPs of the $H_{\varepsilon 22}$ proton of Q62 in pUb (red) and Ub^{WT} (blue). The assignment of the Q62 Hε resonances is from BioMagResBank, BMRB ID: 17769. The pH dependence of the CSP for this proton in pUb was fit to a single pKa value of 7.5 (see also Figure 1h in main text).

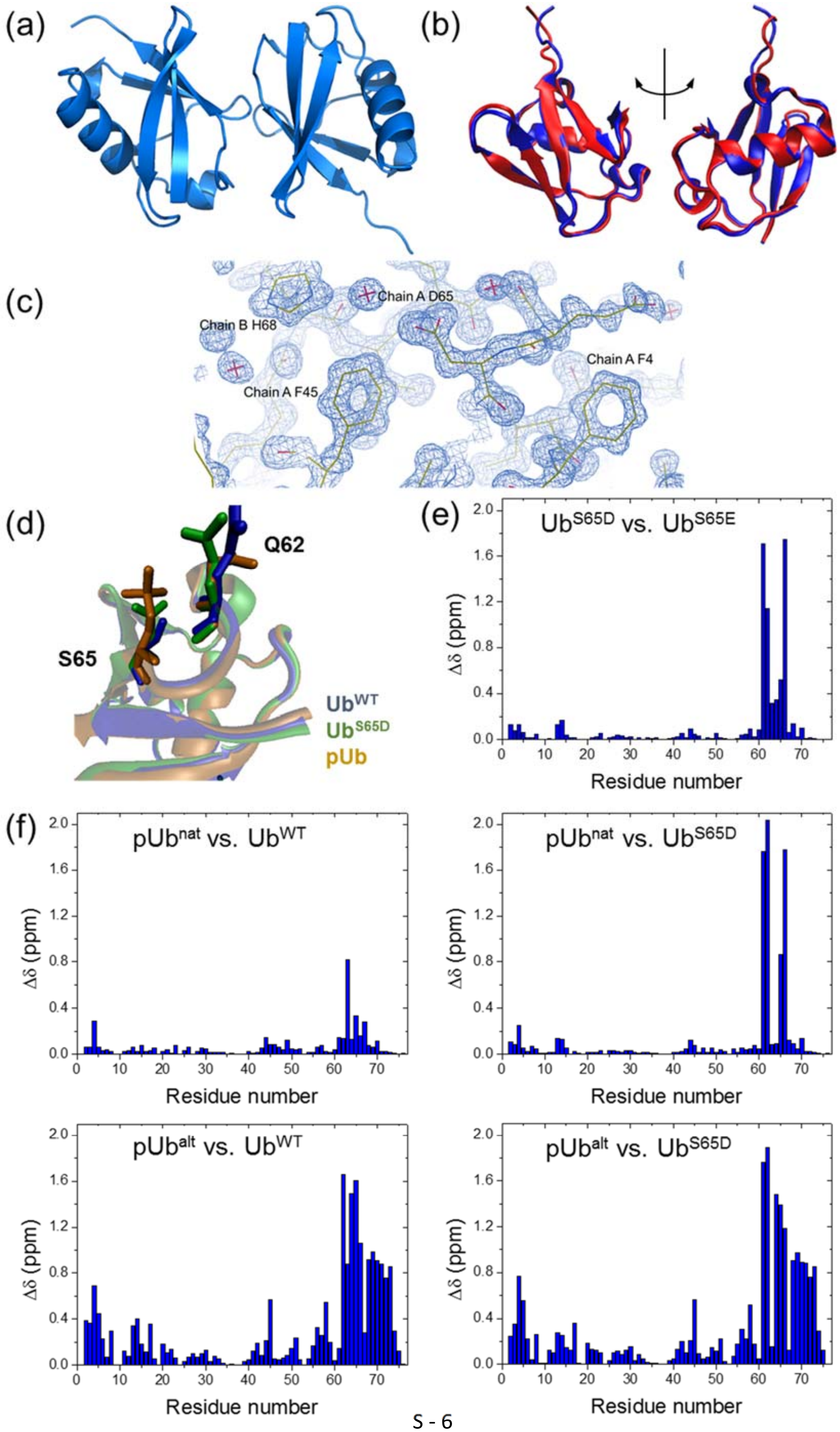

(a)
(b)
(c)
Chain A D65
Chain B H68
Chain A F4
Chain A F45
(d)
Q62
S65
Ub^WT
Ub^S65D
pUb
(e)
Ub^S65D vs. Ub^S65E
Δδ (ppm)
Residue number
(f)
pUb^nat vs. Ub^WT
pUb^nat vs. Ub^S65D
pUb^alt vs. Ub^WT
pUb^alt vs. Ub^S65D
Δδ (ppm)
Residue number

Figure S3. Comparison of the structure and NMR spectra of Ub^{S65D} with Ub^{WT} and pUb. (a) Cartoon view of the two-molecule unit cell in the Ub^{S65D} crystals. (b) Overlay of the structures of Ub^{S65D} (blue) and Ub^{WT} (red). (c) Fragment of the electron density map (2mFo-DFc) for D65 in chain A; the contours correspond to 1.6σ. (d) Overlay of the structures of Ub^{WT}, Ub^{S65D}, and pUb focused on the Ser65-containing loop. (e) CSPs between Ub^{S65D} and Ub^{S65E}. (f) CSPs of the two pUb conformers vs Ub^{WT} or Ub^{S65D}, as indicated.

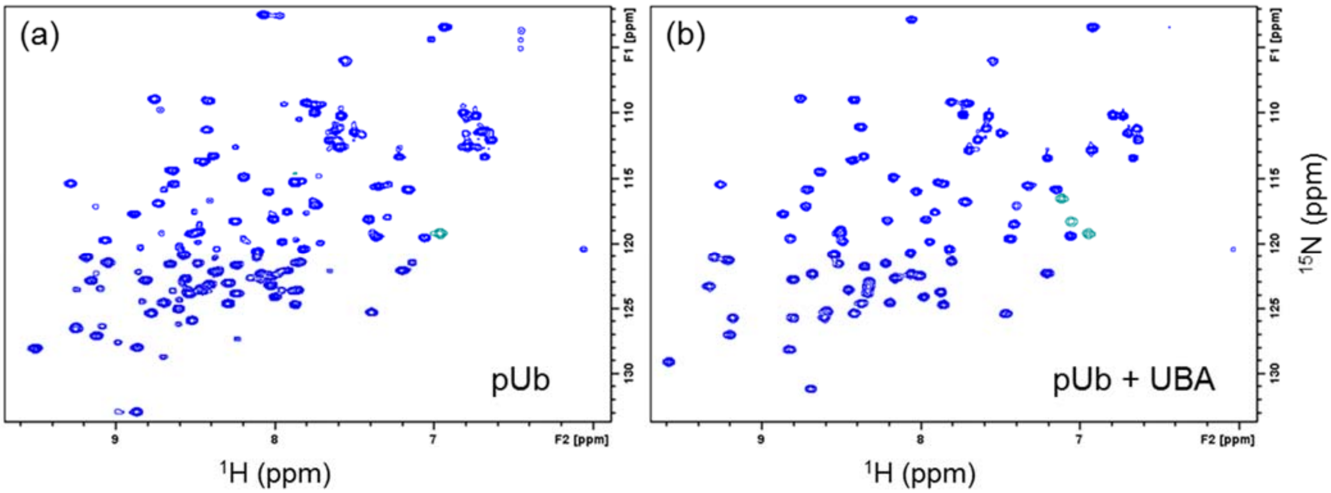

Figure S4. ¹H-¹⁵N SOFAST-HMQC spectra of (a) unbound pUb and (b) pUb at the endpoint of titration with UBA. The NMR signals corresponding to the pUb^{alt} conformer which are present in (a) are entirely absent in (b).

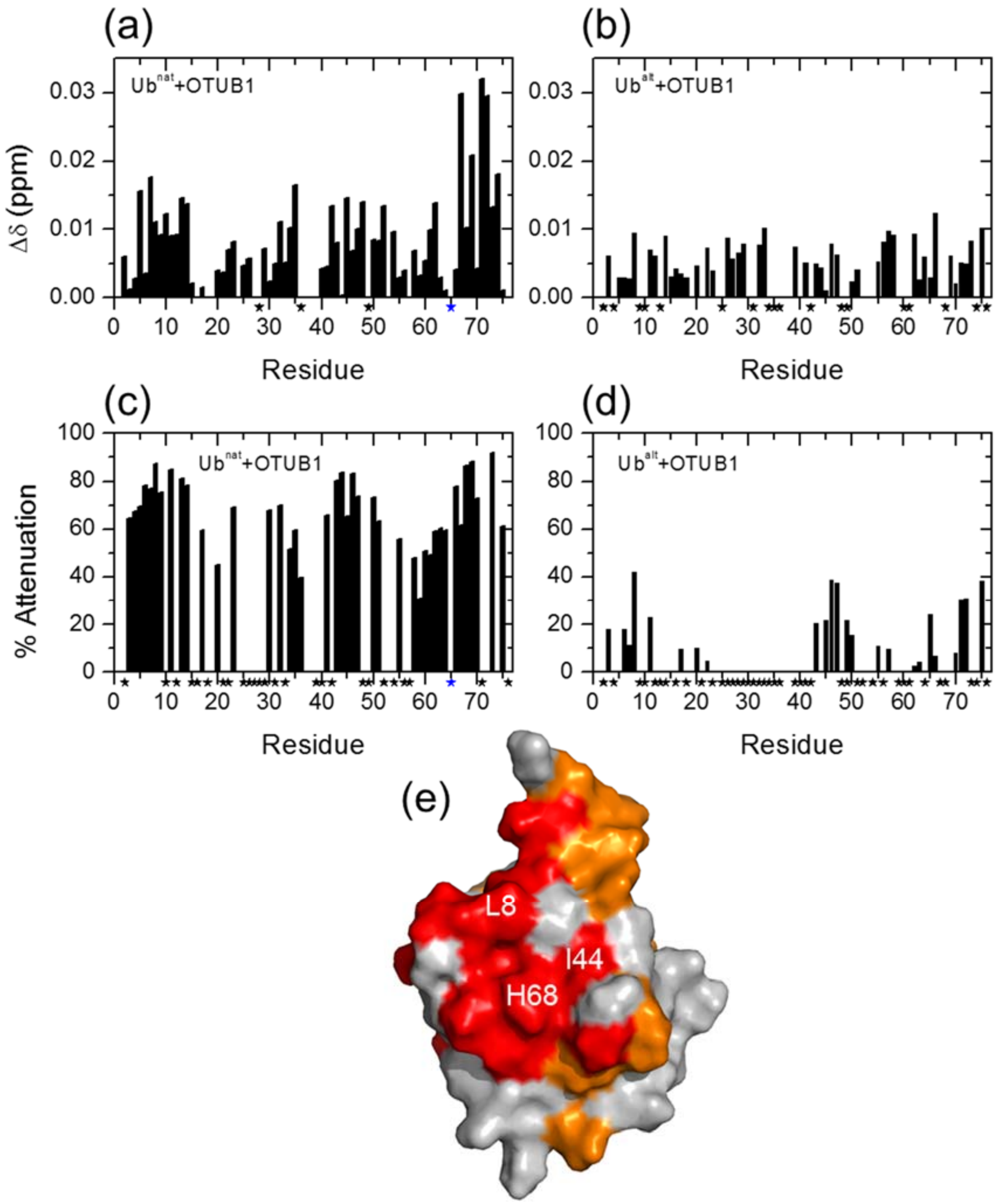

Figure S5. Perturbations in pUb caused by addition of OTUB1 (65 μM pUb + 15 μM OTUB1). Shown as a function of residue number are (a,b) CSPs in (a) pUb^{nat} and (b) pUb^{alt} and (c,d) percent of signal attenuation for (c) pUb^{nat} and (d) pUb^{alt}, calculated as $(1\text{-}I/I_o)\times100\%$, where I_o and I are signal intensities for free pUb and in the presence of OTUB1, respectively. (e) Map of the perturbations in pUb^{nat} on the surface of Ub: residues with $\Delta\delta > 0.013$ ppm are painted orange, those with attenuations > 75% are red. Residues that exhibited both perturbations are painted red. Stars at the bottom of each plot indicate residues that were not included due to signal overlap (black stars) that precluded accurate quantitation or because the signal corresponding to OTUB1 state could not be reliably identified.

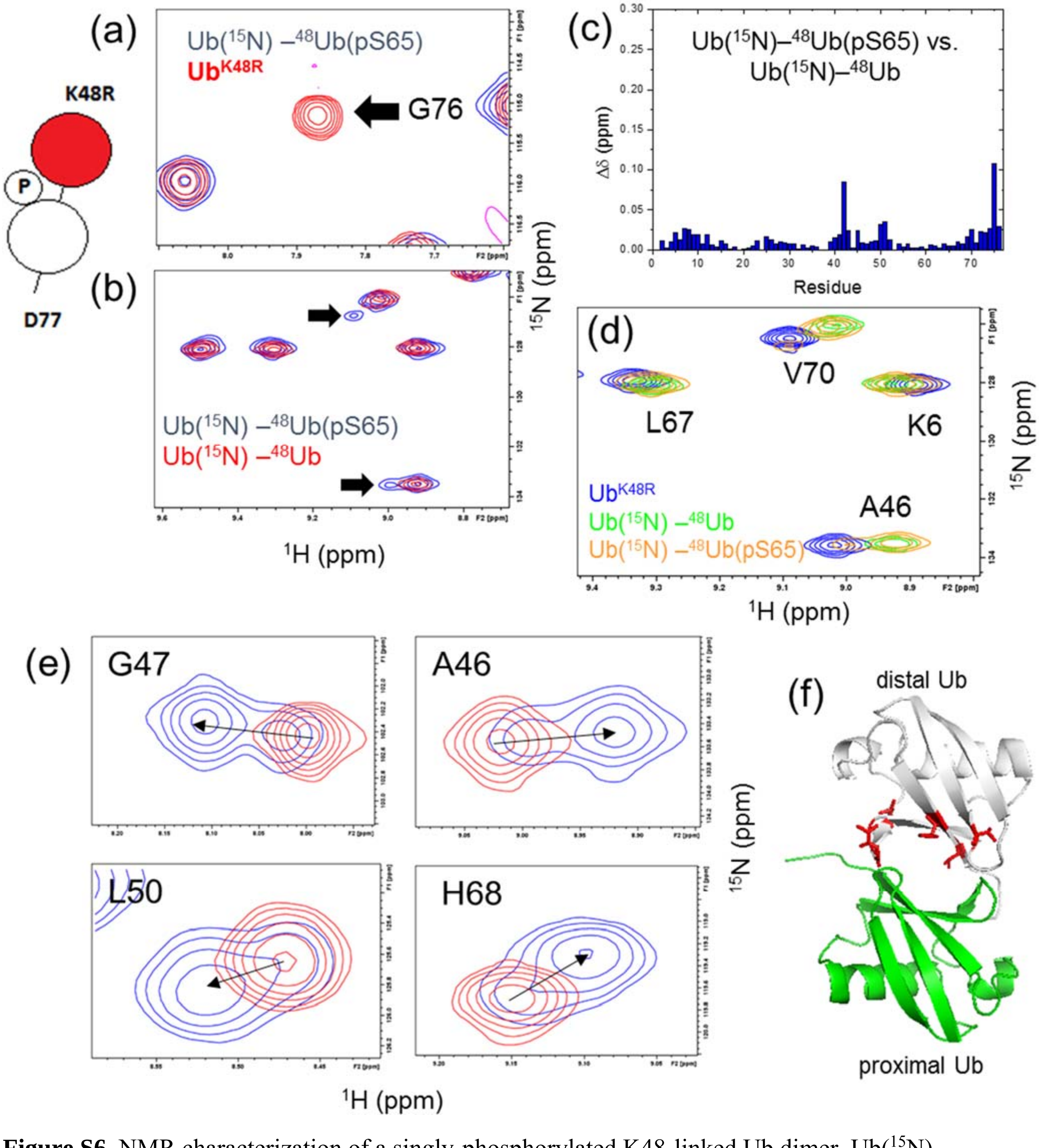

Figure S6. NMR characterization of a singly-phosphorylated K48-linked Ub dimer, Ub(^{15}N)–^{48}Ub(pS65). The dimer is S65-phosphorylated on the proximal Ub and ^{15}N labeled on the distal Ub (see schematic cartoon on the left). (a) Overlay (fragment) of the ^{1}H-^{15}N SOFAST-HMQC spectra of Ub(^{15}N)–^{48}Ub(pS65) (blue) and Ub^{K48R} monomer (red). The absence of the G76 signal (indicated with arrow) in the dimer spectrum indicates that there is no monomeric Ub contaminant. (b) ^{1}H-^{15}N NMR spectrum (fragment) of Ub(^{15}N)–^{48}Ub(pS65) (blue) overlaid with the spectrum of the corresponding unphosphorylated Ub dimer (red). The additional signals in

the phosphorylated dimer (indicated with arrows) do not overlap with unphosphorylated dimer signals, indicating that no unphosphorylated contaminant is present. (c) CSPs of Ub(^{15}N)–^{48}Ub(pS65) versus the distal Ub in the unphosphorylated Ub dimer. The CSPs, especially for the residues involved in the Ub:Ub interface, indicate that the interface is affected by the phosphorylation. (d) Overlay (fragment) of the ^{1}H-^{15}N NMR spectra of Ub(^{15}N)–^{48}Ub(pS65) (gold), distal Ub in unphosphorylated K48-linked Ub dimer (green), and Ub^{K48R} monomer (blue). (e) Overlay of the NMR signals of select residues (indicated) in Ub^{K48R} monomer (red) and in Ub(^{15}N)–^{48}Ub(pS65) (blue), shows a generally linear movement from monomer signals to the "additional signals" to dimer signals. (f) Residues exhibiting the additional signals (e.g., see panel (b)) in the distal Ub are located at the interdomain interface: these residues are mapped (colored red and shown in stick representation) on the structure of K48-linked Ub dimer (PDB ID 1AAR); the distal Ub is colored white.

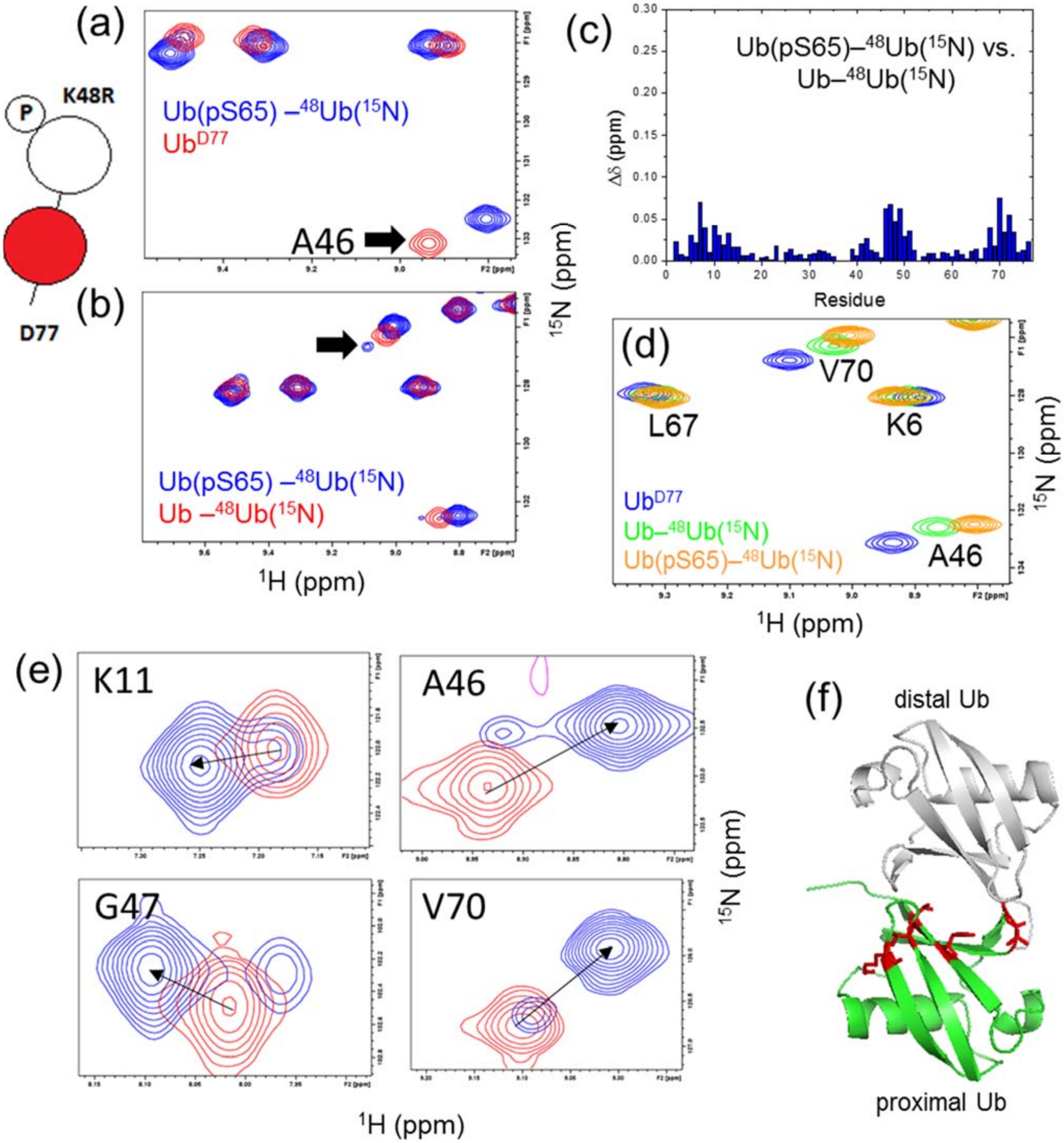

Figure S7. NMR characterization of a singly-phosphorylated K48-linked Ub dimer, Ub(pS65) –^{48}Ub(^{15}N). The dimer is S65-phosphorylated on the distal Ub and ^{15}N labeled on the proximal Ub (see schematic cartoon on the left). (a) Overlay (fragment) of the ^{1}H-^{15}N SOFAST-HMQC spectra of Ub(pS65) –^{48}Ub(^{15}N) (blue) and Ub^{D77} monomer (red). The absence of the A46 signal of monoUb (indicated with arrow) shows lack of monomeric contaminant in the phosphorylated dimer. (b) ^{1}H-^{15}N NMR spectrum (fragment) of Ub(pS65) –^{48}Ub(^{15}N) (blue) overlaid with the spectrum of the corresponding unphosphorylated Ub dimer (red). The additional signals in the

phosphorylated dimer (indicated with arrows) do not overlap with unphosphorylated dimer signals, indicating that no unphosphorylated contaminant is present. (c) CSPs of Ub(pS65) – ^{48}Ub(^{15}N) versus the proximal Ub in the unphosphorylated Ub dimer. The CSPs, especially for the residues involved in the Ub:Ub interface, indicate that the interface is affected by the phosphorylation. (d) Overlay (fragment) of the ^{1}H-^{15}N NMR spectra of Ub(pS65)–^{48}Ub(^{15}N) (gold), proximal Ub in unphosphorylated K48-linked Ub dimer (green), and Ub^{D77} monomer (blue). (e) Overlay of the NMR signals of select residues (indicated) in Ub^{D77} monomer (red) and in Ub(pS65) –^{48}Ub(^{15}N) (blue), shows a generally linear movement from monomer signals to the "additional signals" to dimer signals; G47 appears to be an exception as its signals exhibit a more complicated behavior. (f) Residues exhibiting the additional signals (e.g., see panel (b)) in the proximal Ub are located at the interdomain interface: these residues are mapped (colored red and shown in stick representation) on the structure of K48-linked Ub dimer (PDB ID 1AAR); the proximal Ub is colored green.

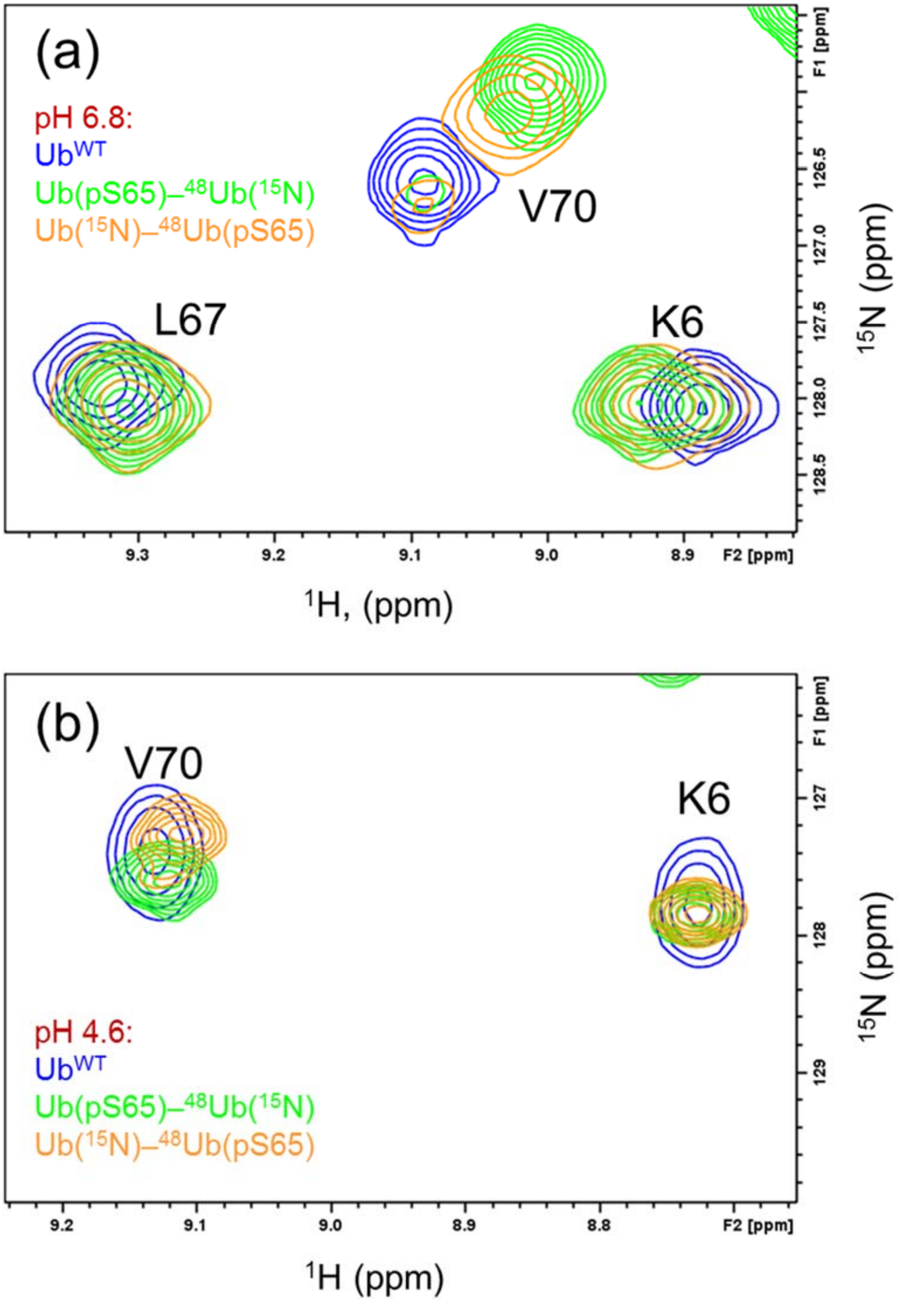

Figure S8. pH-dependence of the noncovalent Ub:Ub interface in phosphorylated K48-linked Ub dimer. Overlay of ^{1}H-^{15}N NMR spectra of the indicated residues in Ub^{WT}(blue), Ub(pS65)–^{48}Ub(^{15}N) (green), and Ub(^{15}N)–^{48}Ub(pS65) (gold) at (a) pH 6.8 and (b) pH 4.6. Shifts of the NMR signals from their positions in monomeric Ub indicate the presence of non-covalent interactions between Ub units in the dimer. These spectra show that, as in the unphosphorylated Ub dimer, the noncovalent Ub:Ub interface (hence the closed state) is present at neutral pH (a) but essentially disappears at low pH (b).

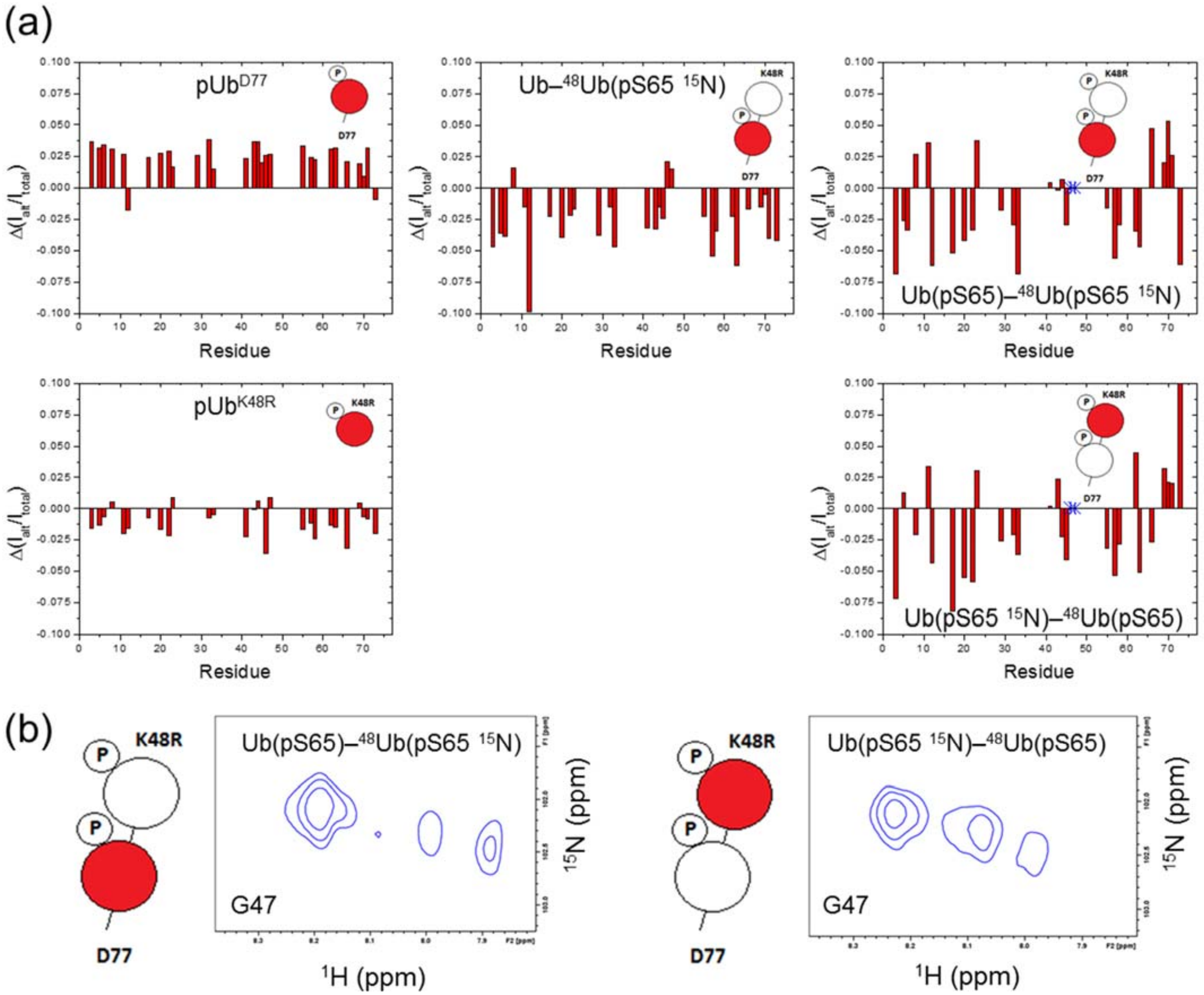

Figure S9. The effect of mutations and interdomain interactions in K48-linked Ub dimer on the equilibrium between the pUb^{nat} and pUb^{alt} conformers of S65 phosphorylated Ub. (a) Difference between the relative population of the pUb^{alt} conformer (calculated as I_{alt}/I_{total}, where $I_{total}=I_{alt}+I_{nat}$) of the indicated monomeric or dimeric-Ub unit and the population of the pUb^{alt} conformer of the phosphorylated monomeric non-mutated Ub (pUb). Blue asterisks indicate residues that could not be used for the analysis due to signal overlap. The cartoons show the Ub variant or Ub unit (colored red) in the dimer that is analyzed. (b) Fragments of ^{1}H-^{15}N NMR spectra showing the presence of three signals for G47 in the indicated phosphorylated Ub units of the doubly-phosphorylated dimer.

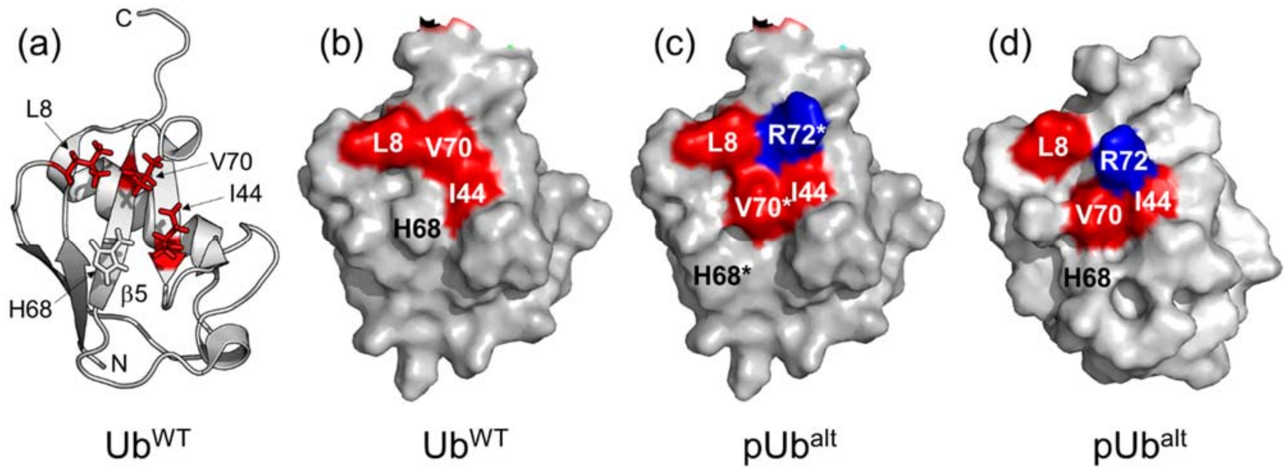

Figure S10. Disruption of the surface hydrophobic patch of Ub in the pUb^{alt} conformer as a result of the retraction of the C terminus caused by the slippage of the β5 strand by two residues. (a) Cartoon representation of the 3-D structure of Ub^{WT} (PDB ID 1D3Z). The canonical hydrophobic patch residues L8, I44, and V70 are shown in stick representation and painted red. Also shown is the side chain of H68. (b) Surface representation of the structure of Ub^{WT}, with the residues L8, I44, V70 painted red. Also indicated is the location of H68. The C-terminal residues G75 nd G76 are not shown. (c) A PyMol-generated model of the expected shifted locations (marked with asterisks) of the side chains of H68, V70, and R72 (painted blue) on the surface of the pUb^{alt} conformer of pUb. This model was obtained by mutating V70 to R, H68 to V, and T66 to H. (d) Surface representation of the structure of the pUb^{alt} conformer of pUb (PDB ID 5XK4) shows a shift in the location of the side chains of H68, V70, and R72 disrupting the canonical surface hydrophobic patch of Ub.
